# Factors Associated With Overuse of Health Care Within US Health Systems: A Cross-sectional Analysis of Medicare Beneficiaries From 2016 to 2018

**DOI:** 10.1001/jamahealthforum.2021.4543

**Published:** 2022-01-14

**Authors:** Jodi B. Segal, Aditi P. Sen, Eliana Glanzberg-Krainin, Susan Hutfless

**Affiliations:** 1 Department of Medicine, Johns Hopkins University School of Medicine, Baltimore, Maryland; 2 Department of Health Policy and Management, Johns Hopkins University Bloomberg School of Public Health, Baltimore, Maryland

## Abstract

**Importance:**

Overuse of health care is a pervasive threat to patients that requires measurement to inform the development of interventions.

**Objective:**

To measure low-value health care use within health systems in the US and explore features of the health systems associated with low-value care delivery.

**Design, Setting, and Participants:**

In this cross-sectional analysis, we identified occurrences of 17 low-value services in 3745 hospitals and affiliated outpatient sites. Hospitals were linked to 676 health systems in the US using the Agency for Healthcare Research and Quality (AHRQ) Compendium of Health Systems. The participants were 100% of Medicare beneficiaries with claims from 2016 to 2018.

**Exposures:**

We identified occurrences of 17 low-value services in 3839 hospitals and affiliated outpatient sites.

**Main Outcomes and Measures:**

Hospitals were linked to health systems using AHRQ’s Compendium of Health Systems. Between March and August 2021, we modeled overuse occurrences with a negative binomial regression model including the year-quarter, procedure indicator, and a health system indicator. The model included random effects for hospital and beneficiary age, sex, and comorbidity count specific to each indicator, hospital, and quarter. The beta coefficients associated with the health system term, normalized, reflect the tendency of that system to use low-value services relative to all other systems. With ordinary least squares regression, we explored health system characteristics associated with the Overuse Index (OI), expressed as a standard deviation where the mean across all health systems is 0.

**Results:**

There were 676 unique health systems assessed in our study that included from 1 to 163 hospitals (median of 2). The mean age of eligible beneficiaries was 75.5 years and 76% were women. Relative to the lowest tertile, health systems in the upper tertile of medical groups count and bed count had an OI that was higher by 0.38 standard deviations (SD) and 0.44 SD, respectively. Health systems that were primarily investor owned had an OI that was 0.56 SD higher than those that were not investor owned. Relative to the lowest tertile, health systems in the upper tertile of primary care physicians, upper tertile of teaching intensity, and upper quartile of uncompensated care had an OI that was lower by 0.59 SD, 0.45 SD, and 0.47 SD, respectively.

**Conclusions and Relevance:**

In this cross-sectional study of US health systems, higher amounts of overuse among health systems were associated with investor ownership and fewer primary care physicians. The OI is a valuable tool for identifying potentially modifiable drivers of overuse and is adaptable to other levels of investigation, such as the state or region, which might be affected by local policies affecting payment or system consolidation.

## Key Points

**Question:**

What features of health care systems in the US are associated with overuse of health care?

**Findings:**

In this cross-sectional study of 676 US health care systems, those that were overusing health care had more beds, had fewer primary care physicians, had more physician practice groups, were more likely to be investor owned, and were less likely to include a major teaching hospital.

**Meaning:**

In-depth exploration of the drivers of health care overuse is needed at the level of health systems as their incentives may not be aligned with high-value care.

## Teaser

This cross-sectional study assesses features of health care systems that are associated with higher provision of low-value or no-value care.

## Introduction

Overuse of health care, or the provision of low-value or no-value care, is consistently identified as contributing to high costs in the US health care system.^1,2,3^ This wasteful care is physically, psychologically, and financially harmful to patients.^4,5,6^ Some interventions that seek to encourage high-value care delivery and limit low-value care are implemented nationally, such as the national coverage determinations of the Medicare program^7^ or bundled payment models.^8,9,10^ Other interventions are delivered locally, within a clinical unit, and are implemented through practice change initiatives. Many of these are motivated by the Choosing Wisely Initiative,^11^ and have had varying effects on reducing low-value care.^12,13,14,15,16^

Health systems may play an important role in the overuse of health care. They balance financial interests when making decisions about strategic consolidations or new service lines, complying with state and federal regulations, and aiming for high-quality care delivery and best patient outcomes. Presently, there is scant quantification regarding low-value health care at the health system level despite the importance of this information for state and federal policy setting.^17^

We previously created an Overuse Index that uses billing codes for diverse clinical services that act as indicators to reflect the latent tendency of a region to overuse health care resources relative to other regions.^18,19,20,21^ Conceptually, the Overuse Index should function like the Consumer Price Index, which uses the average price of a “market-basket” of goods and services to track inflation. In the present work, we have updated the Overuse Index to use *International Statistical Classification of Diseases and Related Health Problems, Tenth Revision (ICD-10)* codes and have adapted it to describe low-value care at the level of health care systems in the United States. Here, we describe US health care systems by their Overuse Index and explore system level factors that are associated with overuse.

## Methods

### Design

This is a serial cross-sectional study approved by the Johns Hopkins Institutional Review Board. The Review Board did not require individual patient consent owing to use of deidentified data.

### Data

We accessed 100% of the inpatient and outpatient claims, and Master Beneficiary Summary files, from Medicare beneficiaries from July 2015 through December 2018 through the Center for Medicare & Medicaid Services’ (CMS) Virtual Research Data Center (VRDC). As required, we did not export pooled information describing between 1 and 10 individuals.

### Identifying Hospitals and Health Systems

The Agency for Healthcare Research and Quality (AHRQ) commissioned a *Compendium of US Health Systems*.^22,23^ For *Compendium* inclusion, health systems needed to include at least 1 non-federal acute care hospital and at least 1 group of physicians connected with the hospital through common ownership or joint management.

The *Compendium*, first generated in 2016, used many data sources including the Healthcare Organization Services and SK&A Healthcare databases, and the American Hospital Association (AHA) survey of hospitals.^22^ The file includes the Center for Medicare & Medicaid Services certification number (CCN) and health system and hospital names and addresses. The hospital linkage file, updated in 2019, more accurately reflects the 2016 relationships, and describes 626 health systems and their hospitals. The linkage information was re-released in 2018, used data from OneKey (owned by IQVIA) and AHA as the primary source of linkage information, and described 637 health systems.

We excluded children’s hospitals, behavioral health centers, psychiatric hospitals, and rehabilitation hospitals by searching for key words in their names. When using the claims from 2016 and 2017, we used the hospital-to-health system linkage information from 2016. When using the 2018 claims, we updated the hospitals’ health systems linkage with the 2018 information.

### Identifying Indicators

We began with the Overuse Index previously described,^20,21^ which included 20 clinically diverse claims-based measures of overuse (indicators), and reviewed the indicators for their continued clinical relevance. We considered additional indicators by reviewing recommendations of the US Preventive Services Task Force and clinical practice guidelines, with the goal of retaining diverse indicators in the Index.

### Identifying Indicator Count for Each Hospital

We identified individuals who were eligible to have each indicator procedure (eg, individuals with mild head trauma could have imaging) with a combination of demographic information, *ICD-10-CM *diagnosis codes, and, rarely, common procedural (CPT) codes (eTable 1 in the Supplement). These eligible individuals were then attributed to the hospital or hospital-associated outpatient facility (clinics or surgical centers) by the CCN associated with the claim having the diagnosis that established their eligibility.

We then identified the subset of individuals, among the eligible, who received the indicator procedure of interest. They were identified by a claim with the relevant *ICD-10-PCS* procedure code or CPT code on any day on which they were eligible. For the indicators for which eligibility was based solely on demographics (eg, women over age 85), the attribution was made based on where the indicator procedure occurred.

We divided the 3 years of data into quarters and included, for any individual, only the first occurrence of a given indicator in a given quarter in a given hospital. An individual could experience multiple different indicators in any quarter-hospital. We conducted our analyses in the spring and summer of 2021.

### Generating the Index

To generate the index, we used a generalized linear mixed model, with a negative binomial distribution, where the dependent variable was the count of the occurrences of the indicator procedures at each hospital in the *i*th quarter (1 to 12), for the *j*th indicator (1 to 17). The offset was the count of individuals eligible in that quarter for that indicator in each hospital. The model included fixed effects for quarter-year, indicator, and health system, as well as random effects for each hospital (Equation 1). The model included patient-level characteristics for the eligible population, specifically mean patient age, proportion of women, and the median count of chronic conditions as generated with CMS’s Chronic Conditions algorithm,^24^ for each hospital-indicator-quarter. The model used the Newton–Raphson optimization option and 1 quadrature point.

C**_ijk_ =**  *Ѡ_i_* + ψ*_j_* + Φ*_k_* + βX_i_ + hospital+ ε*_ij_*

Where *_Ѡi_* is a set of quarter fixed effects, ψ*_j_* is a set of indictor fixed effects, and Φ*_k_* is a set of health system fixed effects, and X_l_ represents a vector of patient characteristics for each hospital-quarter-indicator.

The beta coefficients generated as each health-system’s fixed effects (Φ_k_) are the metrics of interest; they represent composite low-value care use by that health system relative to a reference health system. This measure was standardized to create the Overuse Index as a *Z* score, where the value of the index for the *k*th health system is calculated as in equation 2,

OI*_k_* = (Φ*_k_* − Φ) / SD(Φ)

where OI is the Overuse Index, Φ is the average of the health system fixed effect, and SD(Φ) is the SD across the fixed effects that were estimated in equation 1.

We categorized the health systems according to their standardized Overuse Index into 5 categories based on the *Z* score. Category 1 health systems have an Overuse Index more than 1 SD below the mean, category 2 is between −1 and −0.5 SD below the mean, category 3 is between −0.5 and 0.5 SD of the mean, category 4 is between 0.5 and 1 SD of the mean, and category 5 is more than 1 SD beyond the mean.

### Describing Health Systems

The *Compendium* includes rich information including whether the system has a hospital with a high disadvantaged patient share, or a major teaching hospital.^22^ Other descriptors indicate system-wide uncompensated care burden, teaching intensity, whether the system is predominantly investor-owned, and participation in CMS alternative practice models. We used the characteristics of the health systems in 2018 for description; if the health system existed only in 2016 or 2017, we used the 2016 information. Some variables available in the 2016 data were not available in 2018, and vice versa. For the continuous data, we created indicator variables by tertile as the count data were right-skewed. The health system was attributed to the state where its headquarters were located.

We fit ordinary least squares regression models to estimate independent associations between the Overuse Index and health system characteristics. There were 2 models, the first of which included all health systems (n = 676) and the subset of covariates present in both the 2016 and 2018 *Compendiums*. The second included a smaller set of health systems (n = 486) having the same CCNs in 2016 and 2018, which could be characterized using both the 2016 and 2018 *Compendiums*. We did not impute missing health system characteristics. Characteristics in the final model were chosen to avoid collinearity and to explain the most variance. We included fixed effects for state expecting regional variation.^18,21^ Models were compared with a likelihood ratio test.

### Sensitivity Analyses

We excluded hospitals from contributing to a given indicator if there were fewer than 20 individuals eligible for a given indicator in a hospital, as is done in CMS’s Merit-based Incentive Payment System.^25^ We also tested the relationships between the health system characteristics and the Overuse Index with inclusion of random effects for state using a mixed-effects generalized linear model. The modeling on the VRDC used SAS 9.4; the health system characteristic modeling was done using STATA 15.0.

## Results

### Health Systems

The final data set included 676 health systems. A total of 70 health systems had data in the *Compendium* in 2016 but not in 2018, and 81 systems newly appeared in the *Compendium* data in 2018 (Table 1). The 5 health systems with the most hospitals, consistently between 2016 and 2018, were Catholic Health Initiatives, Ascension Health, Universal Health Services, Community Health Systems, and HCA Health care, each having more than 100 hospitals. One health system in New Hampshire was excluded from modeling owing to missing data. The number of hospitals contributing to these data was 3839. The health systems ranged in size from 1 to 163 hospitals with a median of 2 hospitals (mean of 5.7 hospitals, SD 13.9).

**Table 1.** Characteristics of Included Health Systems

Characteristic	Total No.^a^	Median (range), count
Hospitals	676	
1st tertile	259	1 (1-1)
2nd tertile	233	3 (2-4)
3rd tertile	184	10 (5-183)
Acute care hospitals	676	
1st tertile	277	1 (1-1)
2nd tertile	192	2 (2-3)
3rd tertile	207	8 (4-167)
Beds	676	
1st tertile	226	158 (24-274)
2nd tertile	225	406 (276-639)
3rd tertile	225	1328 (641-36 873)
Discharges	676	
1st tertile	226	6936 (49-12 502)
2nd tertile	225	19 675 (12 504-32 119)
3rd tertile	225	62 140 (32 252-1 843 448)
Medical groups	675	
1st tertile	235	15 (0-25)
2nd tertile	217	40 (26-70)
3rd tertile	223	153 (71-1988)
Physicians	676	
1st tertile	228	95 (50-150)
2nd tertile	223	264 (151-510)
3rd tertile	225	1385 (526-24 955)
Primary care physicians	676	
1st tertile	227	32 (10-54)
2nd tertile	223	96 (55-183)
3rd tertile	225	416 (185-11 090)
Interns and residents	676	
1st tertile	226	0 (0-2)
2nd tertile	225	25 (2-102)
3rd tertile	225	324 (102-2718)
No. of states	676	**Count, No (%)**
1 state		564 (83)
2 states		73 (11)
Multistate		39 (6)
System-wide teaching intensity	676	
Nonteaching		212 (31)
Minor teaching		316 (47)
Major teaching		148 (22)
Has at least 1 hospital with a high disadvantaged patient share	676	226 (33)
Has at least 1 hospital with a high uncompensated care burden	676	226 (33)
Is in the upper quartile of uncompensated care	676	136 (20)
Has at least 1 major teaching hospital	676	223 (33)
Has at least 1 very major teaching hospital	676	104 (15)
Is predominantly investor-owned	676	20 (3)
Has any insurance product	653	214 (33)
Has at least 1 Medicare Advantage plan	546	110 (20)
Has at least 2 Medicaid Managed Care Plan	549	96 (17)
Participates in any accountable care organization contracts	635	283 (44)
Participates in a Medicare bundled payment model	595	287 (48)
Participates in an alternative payment model	606	431 (71)

^a^ Total No. varies depending on availability of information on each characteristic.

### Eligible Patients

By design, the patients who were eligible for each overuse event varied (eTable 2 in the Supplement). The mean age of the beneficiaries eligible for experiencing 1 or more of the overuse events was 73.4 years; 68% were women, and their mean CMS Chronic Conditions count was 7.6.

### Overuse Index

A total of 17 indicators contributed to the Overuse Index. This set included 5 new indicators compared to our earlier work^18,21^; we retired 8 indicators (eTable 3 in the Supplement). The counts of overuse events, for any of the 17 possible events, in a hospital-quarter ranged from 0 to 1414 events. The fewest hospitals contributed to the brain MRI measure (n = 2041) and the most contributed to the hysterectomy measure (n = 3305). Each of the 17 indicators contributed to the Overuse Index proportional to its indicator rate, which might be described as the number of events among all individuals eligible for the indicator event.

The overuse index, before standardization, had a mean of −0.36 (SD 0.40) with a median of −0.30 and range of −3.8 to 0.89 across the 676 systems (Figure). By design, the standardized Overuse Index has a mean of 0 and an SD of 1, with a median of 0.15 with a range from −8.5 to 3.1. There were 101 health systems beyond −1 SD, 67 between −1 and −0.5, 294 between −0.5 and 0.5, 137 between 0.5 and 1.0, and 77 beyond 1 SD. The 214 health systems in the fourth and, particularly, fifth categories can be considered to be health systems that are overusing services relative to the average health system (eTable 4 in the Supplement).

**Figure.**
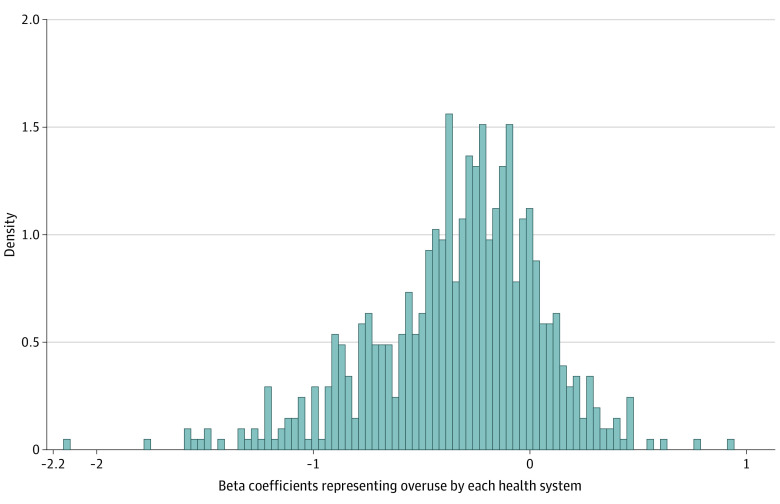
Distribution of Beta Coefficients for Each Health System Removed outlier with beta coefficient = −3.4. The beta coefficients generated as each health system’s fixed effects represent composite low-value care use by that health system relative to a reference health system.

### Health Systems Characteristics Associated With Overuse

In the unadjusted analyses (Table 2), many health system characteristics were strongly associated with higher values on the Overuse Index, including the counts of hospitals, acute care hospitals, and beds. Having a teaching hospital was strongly associated with less overuse. In the multivariable analyses, we observed largely consistent patterns of characteristics that were associated with more or less overuse when looking at the full set of systems (n = 675) and the reduced set (n = 486) (Table 3). Metrics reflecting the size of the health system (ie, number of beds) suggested that the large systems were more often overusing health care relative to small systems; yet, the number of hospitals was not independently associated with higher Overuse Index scores. Health systems with a higher number of medical groups were more likely to be overusing systems, with a dose-response relationship. Strongly negatively associated with overuse was the number of primary care physicians in the system, also demonstrating a dose response relationship; health systems in the upper tertile of primary care physician counts were more than one-half of a SD lower on the Overuse Index than those in the lowest tertile. Health systems that were investor-owned, although few (n = 20), were markedly overrepresented in the highest overuse categories.

**Table 2.** Count and Percentage of Health Systems Within Each Overuse Index Category

Characteristic of 676 health systems	No. (%)				
	>−1	−1 to 0.5	−0.5 to 0.5	0.5-1	>1
Count of hospitals (categories)^a^					
1st tertile	64 (25)	23 (9)	93 (36)	40 (15)	39 (15)
2nd tertile	23 (10)	26 (11)	105 (45)	51 (22)	28 (12)
3rd tertile	14 (8)	18 (10)	96 (52)	46 (25)	10 (5)
Count of acute care hospitals^a^					
1st tertile	69 (25)	24 (9)	99 (36)	44 (16)	41 (15)
2nd tertile	18 (9)	22 (11)	87 (45)	40 (21)	25 (13)
3rd tertile	14 (7)	21 (10)	108 (52)	53 (26)	11 (5)
Count of beds^a^					
1st tertile	48 (21)	23 (10)	92 (41)	33 (15)	30 (13)
2nd tertile	38 (17)	23 (10)	88 (39)	50 (22)	26 (12)
3rd tertile	15 (7)	21 (9)	114 (51)	54 (24)	21 (9)
Count of discharges^b^					
1st tertile	35 (15)	20 (9)	92 (41)	44 (19)	35 (15)
2nd tertile	29 (13)	23 (10)	96 (43)	52 (23)	25 (11)
3rd tertile	37 (16)	24 (11)	106 (47)	41 (18)	17 (8)
Count of medical groups^b^					
1st tertile	47 (20)	21 (9)	84 (36)	46 (20)	37 (16)
2nd tertile	35 (16)	19 (9)	98 (45)	42 (19)	23 (11)
3rd tertile	19 (9)	26 (12)	112 (50)	49 (22)	17 (8)
Count of physicians^b^					
1st tertile	33 (14)	21 (9)	96 (42)	44 (19)	34 (15)
2nd tertile	38 (17)	17 (8)	84 (38)	55 (25)	29 (13)
3rd tertile	30 (13)	29 (13)	114 (51)	38 (17)	14 (6)
Count of primary care physicians^b^					
1st tertile	30 (13)	19 (8)	93 (41)	50 (22)	35 (15)
2nd tertile	39 (17)	19 (8)	86 (38)	50 (22)	29 (13)
3rd tertile	32 (14)	28 (12)	115 (51)	37 (16)	13 (6)
Count of interns and residents					
1st tertile	35 (15)	20 (9)	92 (41)	44 (19)	35 (15)
2nd tertile	29 (13)	23 (10)	96 (43)	52 (23)	25 (11)
3rd tertile	37 (16)	24 (11)	106 (47)	41(18)	17 (8)
System is multistate^b^					
1 state	94 (17)	56 (9)	231 (41)	110 (19)	73 (13)
2 states	3 (4)	7 (10)	43 (59)	16 (22)	4 (5)
More than 2 states	4 (10)	4 (10)	20 (51)	11 (28)	0 (0)
System wide teaching intensity^a^					
No teaching	32 (15)	19 (9)	86 (41)	41 (19)	34 (16)
Minor teaching	28 (9)	26 (8)	146 (46)	79 (25)	37 (12)
Major teaching	41 (28)	22 (15)	62 (42)	17 (11)	6 (4)
System has at least one hospital with a high disadvantaged patient share	43 (19)	20 (9)	95 (42)	44 (19)	24 (11)
System has at least one hospital with a high uncompensated care burden	32 (14)	23 (10)	104 (46)	46 (20)	21 (9)
System is in the upper quartile of uncompensated care	27 (20)	14 (10)	52 (38)	24 (18)	19 (14)
System has at least 1 major teaching hospital^a^	44 (20)	24 (11)	104 (47)	38 (17)	13 (6)
System has at least 1 very major teaching hospital^a^	25 (24)	15 (14)	50 (48)	10 (10)	4 (4)
System is predominantly investor-owned^b^	1 (5)	0 (0)	6 (30)	10 (50)	3 (15)
System has any insurance product	29 (14)	23 (11)	104 (49)	43 (20)	15 (7)
System has at least 1 Medicare Advantage plan	16 (15)	15 (14)	55 (50)	18 (16)	6 (5)
System had a Medicaid managed care contract^c^	19 (20	10 (10)	49 (51)	14 (15)	4 (4)
System participates in any accountable care organization contracts	32 (11)	22 (8)	142 (50)	61(21)	26 (9)
System participates in a Medicare bundled payment model^c^	30 (10)	25 (9)	136 (47)	63 (22)	33 (12)

^a^ Test statistic supports a difference across Overuse categories with *P* value ≤.001.

^b^ Test statistic supports a difference across Overuse categories with a *P* value ≤.01.

^c^ Test statistic supports a difference across Overuse categories with a *P* value ≤.05. Multilevel percentages compared with a χ^2^ test and binary predictors with a Mantel-Haenszel test for linear trend.

**Table 3.** Independent Association of Health System Characteristics With (Standardized) Overuse Index

Characteristic	No.	Difference in Overuse Index^**a**^			
		Model 1 (N = 486)		Model 2 (N = 675)	
			*P* value		*P* value
**Primary care physician category**					
Reference	227				
2nd tertile	223	−0.28^b^	.03^b^	−0.31^b^	<.001^b^
3rd tertile	225	−0.59^b^	.002^b^	−0.60^b^	.008^b^
**Hospital count category**					
Reference	259				
2nd tertile	233	0.19^b^	.05^b^	0.23^b^	.01^b^
3rd tertile	184	0.07	.56	0.13	.36
**Medical group count category**					
Reference	235				
2nd tertile	217	0.29^b^	.02^b^	0.18	.08
3rd tertile	223	0.38^b^	.02^b^	0.27	.08
**Bed count category**					
Reference	226				
2nd tertile	225	0.08	.47	0.19	.65
3rd tertile	225	0.44^b^	.01^b^	0.61^b^	<.001^b^
**Teaching intensity**					
Reference	212				
Minor teaching	316	−0.11	.29	−0.11	.28
Major teaching	148	−0.45^b^	.002^b^	−0.51^b^	<.001^b^
Is primarily investor owned	20	0.56^b^	.01^b^	0.24	.28
Includes a very major teaching hospital	104	−0.31^b^	.01^b^	−0.31^b^	.02^b^
Upper quartile of uncompensated care	136	−0.47^b^	<.001^b^	NA	NA
Participates in a Medicare bundled payment	287	0.11	.17	NA	NA
Participates in a Medicare alternative payment model	431	0.15	.17	NA	NA
Owns a Medicare advantage plan	110	−0.02	.87	NA	NA
Owns a Medicaid managed care plan	96	−0.15	.215	NA	NA
Participates in an accountable care organization contract	283	0.07	.51	NA	NA

NA indicates not applicable.

^a^ Model includes fixed effects for primary state of the health system. All displayed variables are included in the mode, although all variables in Table 2 were evaluated for inclusion. The Overuse Index is standardized so a change of 1 reflects 1 standard deviation change. The reference group is “no” for binary categories; absence of results means that this information was not available for all health systems.

^b^ Statistically significant change with *P* value ≤0.05.

Health systems that were involved in teaching, particularly with the inclusion of a very major teaching hospital, had lower Overuse Index values. Systems in the upper quartile of uncompensated care, relative to those that were not, had an Overuse Index nearly one-half of a SD lower. Participation in CMS programs such as accountable care programs or bundled payment programs was not associated with more or less overuse. Similarly, the ownership of a Medicare Advantage plan or a Medicaid managed care plan was minimally associated with more or less overuse.

### Sensitivity Analyses

When we allowed hospitals to contribute observations only if they had 20 or more eligible people for a given indicator, the results were minimally different (eFigure and eTable 5 in the Supplement). Specifically, 92 of 676 health systems changed by one category (eg, from the third overuse category to the fourth), and only 1 health system changed by 2 categories. The use of random effects models in place of fixed effects models to control for state effects resulted in similar inferences although the sizes of the effects were less extreme (eTable 6 in the Supplement).

## Discussion

Herein, we generated an Overuse Index, with *ICD-10* codes, to report on overuse by individual health systems. This study also demonstrated strong associations between health system factors and overuse that provide additional support for recent observations of similar relationships.^26^ We expect that these findings should further motivate researchers toward designs that allow establishment of causal relationships. Additionally, this study identified novel associations that may generate new testable hypotheses about how system factors affect overuse.

The present study method uses 17 tracers that we consider to be indicators of overuse. We do not consider these to be individually important; we expect that health systems that are overusing these indicator procedures are likely to be globally overusing health services. Although there is variation in overuse across health systems, this variation was less than was demonstrated in our earlier work at a regional level, which used both commercial claims and Medicare claims.^18,21^

The present study methodology is novel and unlike that which is presently most used by others, the MedInsight Health Waste Calculator (from Milliman). The Health Waste Calculator asserts that it measures the absolute amount of care that is wasteful, or likely to be wasteful. Although the contents of the tool are not publicly available, 35 services are described in an article published in 2020.^27^ That tool has been used with state all-payer claims^28^ and to describe trends over time in use of wasteful services by Medicare beneficiaries.^29^ The recent article by Ganguli and colleagues^26^ used some of the Milliman measures.

Like the present work, Ganguli and colleagues^26^ measured overuse of health care by health systems, and there is much concordance in our findings. Ganguli and colleagues measured use of 41 services and averaged the use of 28 services for a summary measure across their 2 years of data. In comparison, the present study used a model to generate the measure of overuse and was thus able to control for differences across hospitals, by adjusting for mean age, sex, and comorbidities in each hospital, in each quarter, for each indicator, over the 3 years of data we used.

Despite the differences in our approaches, there is much agreement in the rankings of the health systems and concordance in factors associated with overuse. Both studies found that the number of physicians in a health system and that the number of primary care physicians is inversely related to overuse in a health system. Both found no significant association of overuse with insurance plan ownership by a health system and no association with an accountable care organization’s presence in a health system. Both found that having a teaching hospital in the health system is inversely associated with overuse. Ganguli and colleagues^26^ found that dual Medicare and Medicaid eligibility within a health system did not meaningfully contribute to overuse. The present study found that health systems in the upper quartile of uncompensated care were much less commonly overusing health systems. We suspect that the variable we used may identify health systems having safety net hospitals, which we expect is different from the dual-eligibility measure.

A related work was published in 2020, when the Lown Institute prepared a measure of waste at the level of 3100 US hospitals.^30,31^ These researchers used 100% of Medicare claims from 2015 through 2017 to measure 13 low-value services, which have much overlap with the 17 that we included. The authors adjusted their observed overuse rates to account for volume differences across hospitals, and then used principal components analysis to reduce the information to one variable that serves as their overuse score. We note that their 10 least overusing teaching hospitals, as listed on their website, are within health systems that we categorized as not highly overusing systems (categories 1, 2, or 3), suggesting concordance of our measures. Similarly, 2 of the hospitals that are ranked by Lown Institute has having a very high likelihood of being overusing hospitals are part of Universal Health Services system, which is a category 4 system with our Overuse Index.

We propose that the Overuse Index has good face validity. The health systems that we expected to be lower in overuse, specifically, those that are integrated health care delivery systems, were indeed lower in overuse: Kaiser Permanente was in category 1. Other systems which are known for their attention to high-value care were also in lower overuse categories: University of Utah Hospitals and Clinics was in category 1 and Intermountain Health Care was in category 3. Other health systems in category 1 are health systems that we suspect are under-resourced as they include large public and safety-net hospitals: these include New York City Health and Hospitals Corporation and Cook County Health and Hospital System. Also supportive of validity, systems that have attracted attention owing to their intensively competitive markets are overusing health systems: UPMC and Allegheny Health Network, both in Pittsburgh, Pennsylvania, are in the fourth and fifth categories, respectively. Systems that are in geographic regions that we previously identified as overusing are prominent in category 5 (systems in Fort Lauderdale and Boynton Beach, Florida, and in Los Angeles, California, and Seattle, Washington.)

The use of AHRQ’s *Compendium* data allowed the present study to explore factors associated with of health system-level overuse. As described above, we demonstrated, once again, that the availability of primary care is associated with less overuse of services. In our earlier work, which focused on measuring overuse regionally, we found that the density of primary care doctors was associated with less overuse of health care, when we examined both commercially insured beneficiaries data and Medicare beneficiaries data.^32,33^ Presently, the measure is the count of primary care physicians in the health system, and we suspect that the density may be inequitable across large systems. This requires further study.

The present study also found a strong relationship between investor ownership of a health system and overuse. There were only 20 investor-owned systems but 12 of the 20 were in category 4 or 5, with only a single one in category 1. The latter was a very small system (25-bed hospital) in Missouri that we suspect may be under-resourced. In 2014, there were federal investigations of several investor-owned health systems with allegations of questionable hospital admissions, procedures, and billings at many hospital systems. Among those investigated were HCA and Health Management Associates that was soon bought by Community Health Systems.^34^ In our work, HCA and Community Health Systems both were category 4 health systems in the years after the investigation.

### Limitations

There are limitations to this approach. Some may challenge the indicators that we chose to include the index; we suspect that fewer indicators might even be sufficient to order health systems similarly. We believe that the inclusion of diverse indicators provides some stability to the index. The investigation of determinants of overuse relies on the validity of the *Compendium* data, which we did not independently verify. Additionally, we used Medicare claims and the included patients were predominantly older adults. We expect, however, that practice patterns within health systems are similar across patients with diverse insurance types—indeed, in our earlier work, the regions that were overusing were largely concordant when we examined commercial claims and Medicare claims.^18,21^

We did not include overuse occurring in office-based ambulatory care for practices that are part of a health system. Again, the goal of the present study was not to count all episodes of overuse; the goal was to create a metric that reflects overuse within a health system relative to other health systems. The concordance with the work of Ganguli and colleagues^26^ essentially demonstrates that the results of this study are correct—one can measure overuse within a health system by focusing measurement on hospital and hospital-based clinics. We strongly suspect that the practice patterns in the hospital and its clinics sets the practice patterns for the whole system and we look forward to exploring this more with qualitative work.

## Conclusions

The findings of this cross-sectional analysis of Medicare beneficiaries and US health systems suggest that the Overuse Index with its publicly available code should be valuable to health systems and be a valuable tool for health services researchers interested in further investigation of drivers of overuse and evaluation of interventions to reduce these harmful practices. In conclusion, we encourage future work to use these classifications of health systems to conduct deeper explorations of determinants of overuse. This may require additional data collection, particularly to better understand the outliers or discordant systems—systems that have characteristics that are associated with overuse and yet are not overusing systems. We anticipate that our software can be run periodically by individual health systems who wish to track their performance over time; it may also be valuable to the Medicare program to test the impact of innovations, although none that we evaluated specifically were associated with more or less overuse (accessible at: https://github.com/susanmhutfless/Overuse-Index-Segal-et-al-Johns-Hopkins-University-ICD-10-Coding).

## Notes

Supplement. eTable 1. Codes for Defining Eligible Populations and Events eTable 2. Characteristics of Individuals Eligible for Each Indicator eTable 3. Comparison of Original and Revised Overuse Index Indicators eTable 4. Health Systems Categorized by Standardized Overuse Index eFigure. Sensitivity Analysis with Exclusion of Low Count Hospitals eTable 5. Tabulation of Health Systems with Inclusion of All Hospitals or Low Counts of Eligible Exclusions eTable 6. Independent Association of Health System Characteristics with (Standardized) Overuse Index with Inclusion of Random Effects for State
