# Errors in Discussion Section and Supplement

**DOI:** 10.1001/jamahealthforum.2022.0132

**Published:** 2022-02-25

The Original Investigation titled “Factors Associated With Overuse of Health Care Within US Health Systems: A Cross-sectional Analysis of Medicare Beneficiaries From 2016 to 2018,”^1^ included an error in the Discussion section when mentioning the work of Ganguli et al.^2^ The proper phrasing is “Ganguli and colleagues measured use of 41 services and averaged the use of 28 services for a summary measure across their 2 years of data.” Additionally, there was an error in eTable 4 in the Supplement. Advocate Aurora Health was incorrectly placed in Category 4 rather than Category 3 due to a typographical error. This has been fixed, and all other information in the Supplement remains correct.
