# Supplementary material for: Factors Associated With Overuse of Health Care Within US Health Systems: A Cross-sectional Analysis of Medicare Beneficiaries From 2016 to 2018
Source: JAMA Health Forum. 2022 Jan 14;3(1):e214543. doi: 10.1001/jamahealthforum.2021.4543 (PMC8903118; doi:10.1001/jamahealthforum.2021.4543)
Supplement: Supplement. — eTable 1. Codes for Defining Eligible Populations and Events eTable 2. Characteristics of Individuals Eligible for Each Indicator eTable 3. Comparison of Original and Revised Overuse Index Indicators eTable 4. Health Systems Categorized by Standardized Overuse Index eFigure. Sensitivity Analysis with Exclusion of Low Count Hospitals eTable 5. Tabulation of Health Systems with Inclusion of All Hospitals or Low Counts of Eligible Exclusions eTable 6. Independent Association of Health System Characteristics with (Standardized) Overuse Index with Inclusion of Random Effects for State [file jamahealthforum-e214543-s001.pdf]

Supplemental Online Content

Segal JB, Sen AP, Glanzberg-Krainin E, Hutfless S. Factors associated with overuse of health care within US health systems: a cross-sectional analysis of Medicare beneficiaries from 2016-2018. JAMA Health Forum. 2022;3(1):e214543. doi:10.1001/jamahealthforum.2021.4543

eTable 1. Codes for Defining Eligible Populations and Events

eTable 2. Characteristics of Individuals Eligible for Each Indicator

eTable 3. Comparison of Original and Revised Overuse Index Indicators

eTable 4. Health Systems Categorized by Standardized Overuse Index

eFigure. Sensitivity Analysis with Exclusion of Low Count Hospitals

eTable 5. Tabulation of Health Systems with Inclusion of All Hospitals or Low Counts of Eligible Exclusions

eTable 6. Independent Association of Health System Characteristics with (Standardized) Overuse Index with Inclusion of Random Effects for State

This supplemental material has been provided by the authors to give readers additional information about their work.

© 2021 Segal et al. *JAMA Health Forum.*

eTable 1. Codes for Defining Eligible Populations and Events

Description	Codes for Eligibility for Overuse Event						Codes Indicating Overuse Event if Concurrent with Eligibility Coding or Otherwise Specified			References
	Inclusion criteria			Exclusion criteria			Inclusion criteria			
	HCPCS	ICD PCs	ICD DX	HCPCs	ICD PCs	ICD DX	HCPCs	ICD PCs	ICD DX	
1.Preoperative chest radiography in the absence of a clinical suspicion for intrathoracic pathology.			Z01.81			J00 to J06<br>J09 to J18<br>J20 to J22<br>J30 to J39<br>J40 to J47<br>J60 to J70<br>J80 to J84<br>J85 to J86<br>J90 to J94	71045<br>71046<br>71010<br>71020	BW03ZZZ		
2. Advanced imaging in acute foot trauma.			S90 to S99				73700 to 73702<br>73718	BQ2J<br>BQ2K<br>BQ2L<br>BQ2M<br>BQ2P<br>BQ2Q<br>BQ2X<br>BQ2Y<br>BQ3J<br>BQ3K<br>BQ3L<br>BQ3M<br>BQ3P<br>BQ3Q		

© 2021 Segal et al. *JAMA Health Forum.*

3. MRI in individuals with mild traumatic brain injury.			S06.0X0A				70551 to 70553	B030Y0Z<br>B030YZZ<br>B030ZZZ		
4. Sinus CT for uncomplicated acute rhinosinusitis.[a]			J01.00 to J01.40<br>J01.90			J32.0 to J32.4<br>J32.8<br>J32.9	70486 to 70488	BN250ZZ<br>BN251ZZ<br>BN25YZZ<br>BN25ZZZ		
5.Abdominal CT with and without contrast							(74150 and 74160) OR 74170	BW2000Z<br>BW2010Z<br>BW20Y0Z		
6. DELETED										
7. MRI of lumbar spine for low back pain.[b]			M54.5			C00 to C43<br>C45 to C96<br>M46.2 to M46.5<br>S34	(72148 and 72149) OR 72158	BR39Y0Z<br>BR39YZZ<br>BR39ZZZ		
8. Traction for low back pain.			M54.5				97012	2W65X0Z<br>2W65XZZ		

© 2021 Segal et al. *JAMA Health Forum.*

9. Hysterectomy for benign disease.						C53 to C56	58150<br>58152<br>58180<br>58200<br>58210<br>58260<br>58262<br>58263<br>58267<br>58270<br>58275<br>58280<br>58285<br>58290 to<br>59294<br>58541 to<br>58544<br>58548<br>58550<br>58552 to<br>58554<br>58570 to<br>58573	0UT94ZL<br>0UT90ZL<br>0UT94ZZ<br>0UT90ZZ<br>0UT9FZL<br>0UT9FZZ<br>0UT97ZL<br>0UT98ZL<br>0UT97ZZ<br>0UT98ZZ<br>0UT44ZZ<br>0UT94ZZ<br>0UT40ZZ<br>0UT90ZZ<br>0UT44ZZ<br>0UT9FZZ<br>0UT47ZZ<br>0UT48ZZ<br>0UT97ZZ<br>0UT98ZZ<br>0UT90ZZ<br>0UT94ZZ<br>0UT90ZL<br>0UT90ZZ<br>0UT94ZL<br>0UT94ZZ<br>0UT97ZL<br>0UT97ZZ<br>0UT98ZL<br>0UT98ZZ<br>0UT9FZL<br>0UT9FZZ		

© 2021 Segal et al. *JAMA Health Forum.*

10. Spinal fusion[c] (restricted to the back pain population from #7)	(as above)		M54.5			C00 to C43<br>C45 to C96<br>M46.2 to M46.5<br>S34	22558<br>22612<br>22630<br>22633	0SG0070<br>0SG0071<br>0SG007J<br>0SG00A0<br>0SG00AJ<br>0SG00J0<br>0SG00J1<br>0SG00JJ<br>0SG00K0<br>0SG00K1		
11. Meniscectomy in patients with degenerative joint disease of the knee.			M17	V00-W19	0SRC06A<br>0SRC06Z<br>0SRC07Z<br>0SRC0EZ<br>0SRC0J9<br>0SRC0JA<br>0SRC0JZ<br>0SRC0KZ<br>0SRC0L9<br>0SRC0LA<br>0SRC0LZ<br>0SRC0M9<br>0SRC0MA<br>0SRC0MZ<br>0SRC0N9<br>0SRC0NA<br>0SRC0NZ<br>0SRD069<br>0SRD06A<br>0SRD06Z<br>0SRD07Z<br>0SRD0EZ<br>0SRD0J9		29881<br>27332<br>27333<br>27403<br>29868<br>29880<br>29881 to 29883	0SBC4ZZ<br>0SBD4ZZ		

© 2021 Segal et al. *JAMA Health Forum.*

					0SRD0JA<br>0SRD0JZ<br>0SRD0KZ<br>0SRD0L9<br>0SRD0LA<br>0SRD0LZ<br>0SRD0M9<br>0SRD0MA<br>0SRD0MZ<br>0SRD0N9<br>0SRD0NA<br>0SRD0NZ					
12. Nasal endoscopy for sinusitis diagnosis.[a]			J01.00 to<br>J01.40<br>J01.90			J32.0 to<br>J32.4<br>J32.8<br>J32.9	31231<br>31233<br>31235	09JK0ZZ<br>09JK3ZZ<br>09JK4ZZ<br>09JK8ZZ<br>09JKXZZ<br>09JY0ZZ<br>09JY3ZZ<br>09JY4ZZ<br>09JY8ZZ<br>09JYXZZ		
13.PAP smear in women over 65 years.[d]							Q0091<br>G0123-4<br>G0141-8<br>P3000<br>P3001<br>Q0091		Z01.411<br>Z01.419<br>Z12.4<br>Z12.72<br>Z12.79<br>Z12.89<br>Z11.51	

© 2021 Segal et al. *JAMA Health Forum.*

14.Screening mammography in women 85 years and older.[e]							G0202<br>77067<br>77063			
15.Screening for colorectal cancer in adults 80 years and older.[f]							45378 to 45398<br>G0105<br>G0121<br>45330 to 45350<br>88305<br>G0104		Z12.11	
16.Screening for asymptomatic carotid artery stenosis in the adult population over age 80 years.						I63 to I69<br>R55	93880<br>3100F	B345ZZ3<br>B345ZZZ<br>B348ZZ3<br>B348ZZZ		
17. Routine monitoring of digoxin in patients with congestive heart failure.			I50			T46.0X1 to 5	80162			

© 2021 Segal et al. *JAMA Health Forum.*

18.EEG monitoring in individuals presenting with syncope.			R55				3650F<br>95812<br>95813<br>95816<br>95819<br>95822<br>95827<br>95950<br>95951<br>95953<br>95956	4A0034Z<br>4A0074Z<br>4A0084Z<br>4A00X4Z<br>4A1034Z<br>4A1074Z<br>4A1084Z<br>4A10X4Z<br>4A0034Z<br>4A0074Z<br>4A0084Z<br>4A00X4Z<br>4A1034Z<br>4A1074Z<br>4A1084Z<br>4A10X4Z		

Footnotes:

CT=Computed tomography, Dx=diagnosis code, EEG=electro to encephalography, HCPCS=Healthcare Common Procedure Coding System, ICD= International Classification of Disease MRI=Magnetic resonance imaging; PC=procedure code PET=Proton emission tomography

a. Eligible are people WITH the inclusionary ICD to CM and WITHOUT the exclusionary ICD to CM in the preceding 180 days

b. People WITH the inclusionary ICD to CM are eligible for overuse event unless they have an EXCLUSIONARY code in the next 60 days

c. People WITHOUT the exclusionary ICD to CM are eligible for overuse event in the next 90 days; single level fusion procedure

d. Women over 65.0 years

e. Women over 80.0 years

f. Men and women over 80.0 years

g. People WITHOUT the exclusionary ICD to CM are eligible for overuse event in the next 30 days

The exclusion criteria for the eligible population are also applied to the population having the event of interest on the day of the event of interest.

© 2021 Segal et al. *JAMA Health Forum.*

eTable 2. Characteristics of Individuals Eligible for Each Indicator

**Indicator #**	**1**	**2**	**3**	**4**	**5**	**6**	**7**	**8**	**9**
**Name**	**Preoperative Chest X-ray**	**Foot imaging**	**MRI In Mild Traumatic Brain Injury**	**Sinus CT in Rhinosinusitis**	**Abdominal CT with/without**	(Dropped)	**MRI of Lumbar Spine**	**Traction for Low Back Pain**	**Hysterectomy for Benign Disease**
Hospitals contributing information	3390	3117	2041	2845	2795		3135	3136	3305
Eligible count (12 quarters)	5211908	1299225	81883	589608	492778		4846881	4866919	112679345
Event count (12 quarters)	1224720	31294	1858	21203	170528		432435	20015	47365
Mean age at eligibility, yr	71	67	69	67	71		69	69	72
Percent female among eligible	55	63	66	65	55		61	61	100
Median comorbidity count among eligible	6.4	6.8	7.0	6.1	7.0		6.9	6.9	6.7

**Indicator #**	**10**	**11**	**12**	**13**	**14**	**15**	**16**	**17**	**18**
**Name**	**Spinal fusion, single level**	**Meniscectomy in Patients with DJD**	**Nasal Endoscopy for Sinusitis**	**Pap Smear over 65 Years**	**Mammo-graphy over 85 Years**	**Colonoscopy over 80 Years**	**Screening for Carotid Artery Stenosis over 80 years**	**Digoxin for monitoring purposes**	**EEG Monitoring In Syncope**
Counts of hospitals contributing information	3136	3134	2843	3300	3258	3203	3203	3052	3100
Eligible count (12 quarters)	4866865	3127802	589840	92988093	28050302	42257476	42243254	678682	2017605
Event count	925	40541	2209	233723	956651	904365	125513	128763	57027

© 2021 Segal et al. *JAMA Health Forum.*

(12 quarters)									
Mean age at eligibility, yr	69	72	67	76	86	86	86	74	74
Percent female among those eligible	61	66	65	100	100	63	63	48	56
Median comorbidity count among those eligible	6.9	6.6	6.1	5.9	9.4	9.4	9.4	11	8.0

© 2021 Segal et al. *JAMA Health Forum.*

eTable 3. Comparison of Original and Revised Overuse Index Indicators

ICD-10 Based Overuse Index	ICD-9 Based Overuse Index	Reference	Rationale for Removal
Abdominal CT with and without contrast	Abdominal CT with and without contrast	Quality Net[1]	NA
Sinus CT for uncomplicated acute rhinosinusitis.	Sinus CT for uncomplicated acute rhinosinusitis.	Choosing Wisely[2]	NA
Electroencephalogram (EEG) monitoring in individuals presenting with syncope	Electroencephalogram (EEG) monitoring in individuals presenting with syncope	National Health Service, UK[3]	NA
Hysterectomy for benign disease.	Hysterectomy for benign disease	National Guidelines Clearinghouse[4]	NA
Laminectomy and/or spinal fusion	Laminectomy and/or spinal fusion	National Guidelines Clearinghouse[4]	NA
Magnetic Resonance Imaging (MRI) in individuals with traumatic brain injury	Magnetic Resonance Imaging (MRI) in individuals with traumatic brain injury	National Quality Forum[5]	NA
MRI Lumbar Spine for Low Back Pain	MRI Lumbar Spine for Low Back Pain	Quality Net[1]	NA
Nasal endoscopy for sinusitis diagnosis	Nasal endoscopy for sinusitis diagnosis	The Alternative Quality Contract[6]	NA
Preoperative chest radiography in the absence of a clinical suspicion for intrathoracic pathology	Preoperative chest radiography in the absence of a clinical suspicion for intrathoracic pathology	Qaseem et al[7]	NA
Routine monitoring of digoxin in patients with congestive heart failure.	Routine monitoring of digoxin in patients with congestive heart failure	National Health Service, UK[3]	NA
Traction for low back pain.	Traction for low back pain	Institute of Medicine[8]	NA
Screening for asymptomatic carotid artery stenosis in adults age 80 years and older **(Modified to include age restriction)**	Screening for asymptomatic carotid artery stenosis (CAS) in the general adult population	US Preventative Services Task Force[9]	NA
Advanced imaging in acute foot trauma **(NEW)**	NA	ACR Appropriateness Criteria Acute Trauma to the Foot[10]	NA
Meniscectomy in patients with degenerative joint disease of the knee **(NEW)**	NA	Ample trial evidence	NA

© 2021 Segal et al. *JAMA Health Forum.*

PAP smear in women 65 years and older (**NEW)**	NA	US Preventative Services Task Force[11]	NA
Screening for colorectal cancer in adults 80 years and older **(NEW)**	NA	US Preventative Services Task Force [12] (modified)	NA
Screening mammography in women 80 years and older **(NEW)**	NA	US Preventative Services Task Force [13] (modified)	NA
Removed	Stress echocardiography for detection of CAD/risk assessment in symptomatic or ischemic equivalent acute chest pain	American College of Cardiology Foundation, American Society of Echocardiography, American Heart Association, American Society of Nuclear Cardiology, Heart Failure Society of America, Heart Rhythm Society, Society for Cardiovascular Angiography and Interventions, Society of Critical Care Medicine, Society of Cardiovascular Computed Tomography, Society for Cardiovascular Magnetic Resonance American College of Chest Physicians1	Challenging to operationalize
Removed	Fiberoptic laryngoscopy for patients with a diagnosis of sinusitis	The Alternative Quality Contract	Too similar to an included indicator.
Removed	More than one emergency department visit in last 30 days of life	National Quality Forum	Challenging to operationalize
Removed	Serological tests for helicobacter pylori	National Health Service, UK	Not paid for by Medicare
Removed	Positron emission tomography (PET), Computed Tomography (CT), and radionuclide bone scans in individuals with low risk prostate cancer	Choosing Wisely	In ability to identify low risk prostate cancer with claims

© 2021 Segal et al. *JAMA Health Forum.*

Removed	Performing tumor marker studies in asymptomatic women with previously treated breast cancer	Qaseem et al	Rare
Removed	Diagnostic tests, such as immunoglobulin G (IgG) testing or an indiscriminate battery of immunoglobulin E (IgE) tests, in the evaluation of allergy	Choosing Wisely	Rare
Removed	Thorax CT Use of Contrast Material	Quality Net	Similar to an included indicator

References for eTable 3

1. QualityNet. Imaging efficiency measures. Available at: https://www.qualitynet.org/dcs/ContentServer?cid=1228772297509&pagename=QnetPublic%2FPage%2FQnetTier3&c=Page. Accessed July 9, 2018
2. ABIM Foundation. Choosing Wisely. 2018. Available at: http://www.choosingwisely.org/clinician-lists/. Accessed July 9, 2018
3. National Institute for Health and Care Excellence (NICE). Do not do. Available at: https:/ www.nice.org.uk/about/what we-do/into-practice. Accessed July 9, 2018.
4. Agency for Healthcare Research and Quality. National Guideline Clearinghouse. Available at: http://www.guideline.gov/index.aspx. Accessed July 9, 2018.
5. National Quality Forum. Quality Positioning System. Available at: https://www.qualityforum.org/QPS/. Accessed July 9, 2018.
6. Blue Cross Blue Shield of Massachusetts. The Alternative QUALITY Contract. Available at: http://www.bluecrossma.com/visitor/pdf/alternative-quality-contract.pdf. Accessed July 9, 2018.
7. Qaseem A, Alguire P, Dallas P, et al. Appropriate use of screening and diagnostic tests to foster high-value, cost-conscious care. Ann Intern Med. 2012;156:147–149.
8. Institute of Medicine. Knowing What Works in Healthcare: A Roadmap for the Nation. Washington, DC: The National Academies Press; 2008.
9. US Preventative Services Taskforce. Screening for carotid artery stenosis. 2007. Available at: http://www.uspreventiveservicestaskforce.org/uspstf/uspsacas.htm. Accessed July 9, 2018.
10. Expert Panel on Musculoskeletal Imaging, Gorbachova T, Chang EY, Ha AS, Amini B, Dorfman SR, Fox MG, Khurana B, Klitzke A, Lee KS, Mooar PA, Shah KH, Shah NA, Singer AD, Smith SE, Taljanovic MS, Thomas JM, Kransdorf MJ. ACR Appropriateness Criteria® Acute Trauma to the Foot. J Am Coll Radiol. 2020 May;17(5S):S2-S11.
11. US Preventative Services Taskforce. Cervical Cancer: Screening 2019. Available at: https://www.uspreventiveservicestaskforce.org/uspstf/recommendation/cervical-cancer-screening
12. US Preventative Services Taskforce. Colorectal Cancer: Screening 2020. Available at: https://www.uspreventiveservicestaskforce.org/uspstf/recommendation/colorectal-cancer-screening (site updated 2021)

© 2021 Segal et al. *JAMA Health Forum.*

eTable 4. Health systems Categorized by Standardized Overuse Index

We aimed to measure low-value health care use within health systems in the United States and explore features of the health systems associated with low-value care delivery.

With claims from 100% of Medicare beneficiaries from 2016-2018, we identified occurrences of 17 low-value services in 3745 hospitals and affiliated outpatient sites. Hospitals were linked to health systems using AHRQ's Compendium of Health Systems.

We modeled overuse occurrences with a negative binomial regression model including the year-quarter, procedure indicator, and a health system indicator. The model included random effects for hospital and beneficiary age, sex, and comorbidity count specific to each indicator, hospital, and quarter. The beta-coefficients associated with the health system term, normalized, reflects the tendency of that system to use low-value services relative to all other systems.

To describe the health systems, we categorized them according to their standardized Overuse Index and placed them in five categories. Category 1 health systems have an Overuse Index more than 1 SD below the mean, category 2 is between -1 and -0.5 SD below the mean, category 3 is between -5 and 5 SD of the mean, category 4 is between 0.5 and 1 SD of the mean, and category 5 is more than 1 SD beyond the mean. On the following pages, we list the health systems in each category alphabetically by state and by name. The health system ID is that assigned by the Agency for Healthcare Research and Quality and the city and state are for the home office of the health system.

© 2021 Segal et al. *JAMA Health Forum.*

Category 1

Health System ID	Name	City	State	Standardized Overuse Index
HSI00001145	Unity Health	Searcy	AR	-1.41
HSI00000615	Maricopa Integrated Health System	Phoenix	AZ	-2.18
HSI00000018	Alameda Health System	Oakland	CA	-1.04
HSI00000053	Arrowhead Regional Medical Center	Colton	CA	-1.09
HSI00000270	Contra Costa Health Services	Martinez	CA	-1.78
HSI00000345	Enloe Medical Center	Chico	CA	-1.20
HSI00000503	Huntington Memorial Hospital	Pasadena	CA	-1.29
HSI00000536	Kaiser Permanente	Oakland	CA	-1.09
HSI00000593	Los Angeles County Health Services Department	Los Angeles	CA	-1.85
HSI00000723	Natividad Medical Center	Salinas	CA	-1.36
HSI00000880	Rideout Health Group	Yuba City	CA	-1.06
HSI00000885	Riverside University Health System	Moreno Valley	CA	-1.28
HSI00001351	San Francisco Health Network	San Francisco	CA	-2.43
HSI00001223	Ventura County Health Care Agency	Ventura	CA	-1.04
HSI00000307	Denver Health	Denver	CO	-2.28
HSI00000720	National Jewish Health	Denver	CO	-1.29
HSI00000201	Charlotte Hungerford Hospital	Torrington	CT	-1.40
HSI00001176	University of Miami Health System	Miami	FL	-1.20
HSI00000412	Grady Health System	Atlanta	GA	-3.01
HSI00001287	Broadlawns Medical Center	Des Moines	IA	-2.78
HSI00001172	University of Iowa Hospitals and Clinics	Iowa City	IA	-1.09
HSI00000148	CGH Medical Center	Sterling	IL	-1.02
HSI00000274	Cook County Health and Hospital System	Chicago	IL	-2.65
HSI00000539	Katherine Shaw Bethea Hospital	Dixon	IL	-2.12
HSI00001171	University of Illinois Medical Center	Chicago	IL	-1.27
HSI00000349	Eskenazi Health	Indianapolis	IN	-2.79
HSI00000914	Saint Claire Regional Medical Center	Morehead	KY	-1.83
HSI00000559	LSU Healthcare Services Division	Baton Rouge	LA	-1.60
HSI00000131	Boston Medical Center	Boston	MA	-2.28
HSI00000156	Cambridge Health Alliance	Cambridge	MA	-1.58

© 2021 Segal et al. *JAMA Health Forum.*

HSI00000287	Covenant Health Systems	Tewksbury	MA	-1.34
HSI00001112	Tufts Medical Center	Boston	MA	-1.31
HSI00000380	Frederick Regional Health System	Frederick	MD	-1.43
HSI00000197	Central Maine Healthcare Corporation	Lewiston	ME	-1.46
HSI00000330	Eastern Maine Healthcare Systems	Brewer	ME	-1.57
HSI00000610	MaineGeneral Health	Augusta	ME	-1.34
HSI00000611	MaineHealth	Portland	ME	-1.01
HSI00000680	Mid Coast Health Services	Brunswick	ME	-1.18
HSI00001274	York Hospital	York	ME	-1.82
HSI00000676	Metro Health	Wyoming	MI	-1.22
HSI00000350	Essentia Health	Duluth	MN	-1.06
HSI00000473	Hennepin Health Care System	Minneapolis	MN	-2.44
HSI00000765	Northfield Hospital and Clinics	Northfield	MN	-1.03
HSI00000789	Olmsted Medical Center	Rochester	MN	-1.88
HSI00000937	Saint Lukes Hospital of Duluth	Duluth	MN	-1.45
HSI00001264	Winona Health	Winona	MN	-1.96
HSI00000225	Citizens Memorial Healthcare	Bolivar	MO	-1.40
HSI00000804	Ozarks Community Hospital Health System	Springfield	MO	-4.42
HSI00000655	Memorial Hospital at Gulfport	Gulfport	MS	-1.73
HSI00000135	Bozeman Deaconess Hospital	Bozeman	MT	-1.27
HSI00000943	St. Peters Health	Helena	MT	-1.39
HSI00000162	Cape Fear Valley Health System	Fayetteville	NC	-1.34
HSI00001235	Wake Forest University Baptist Medical Center	Winston Salem	NC	-1.03
HSI00000165	Capital Region Health Care	Concord	NH	-1.89
HSI00000188	Catholic Medical Center	Manchester	NH	-1.89
HSI00000338	Elliot Health System	Manchester	NH	-1.45
HSI00000385	Frisbie Memorial Hospital	Rochester	NH	-2.06
HSI00001247	Wentworth Douglass Hospital	Dover	NH	-1.28
HSI00000843	Presbyterian Healthcare Services	Albuquerque	NM	-1.04
HSI00001125	UNM Hospitals	Albuquerque	NM	-1.17
HSI00001160	University Medical Center of Southern Nevada	Las Vegas	NV	-1.62
HSI00000044	ArchCare	New York	NY	-8.48
HSI00000087	Bassett Healthcare Network	Cooperstown	NY	-1.09
HSI00000140	BronxCare Health System	Bronx	NY	-2.13
HSI00000515	Interfaith Medical Center	Brooklyn	NY	-2.14
HSI00000550	Kingsbrook Jewish Medical Center	Brooklyn	NY	-1.35
HSI00000642	Medisys Health Network	Jamaica	NY	-1.02

© 2021 Segal et al. *JAMA Health Forum.*

HSI00000730	New York City Health and Hospitals Corporation	New York	NY	-3.52
HSI00000774	NuHealth	East Meadow	NY	-3.04
HSI00000910	SBH Health System	Bronx	NY	-2.99
HSI00000903	SUNY Downstate Medical Center	Brooklyn	NY	-1.97
HSI00000008	Adena Health System	Chillicothe	OH	-1.27
HSI00000490	Holzer Health System	Gallipolis	OH	-1.07
HSI00001043	Summa Health System	Akron	OH	-1.29
HSI00001083	The Metrohealth System	Cleveland	OH	-2.11
HSI00001090	The University of Toledo Medical Center	Toledo	OH	-1.61
HSI00001118	UC Health	Cincinnati	OH	-1.75
HSI00000912	Saint Charles Health System	Bend	OR	-1.75
HSI00000947	Salem Health	Salem	OR	-1.19
HSI00000951	Samaritan Health Services	Corvallis	OR	-1.34
HSI00000868	Regional Health	Rapid City	SD	-1.02
HSI00000870	Regional One Health	Memphis	TN	-2.85
HSI00001222	Vanderbilt University Medical Center	Nashville	TN	-1.16
HSI00000451	Harris Health System	Houston	TX	-2.10
HSI00001063	JPS Health Network	Fort Worth	TX	-1.59
HSI00000298	Parkland Health and Hospital System	Dallas	TX	-1.45
HSI00001184	University of Utah Hospitals and Clinics	Salt Lake City	UT	-1.52
HSI00000063	Augusta Health	Fishersville	VA	-1.23
HSI00001308	Gifford Health Care	Randolph	VT	-2.13
HSI00000835	Porter Medical Center, Inc	Middlebury	VT	-2.32
HSI00000266	Confluence Health	Wenatchee	WA	-1.39
HSI00000353	Evergreen Health	Kirkland	WA	-1.78
HSI00000714	Multicare Health System	Tacoma	WA	-1.03
HSI00000229	Olympic Medical Center	Port Angeles	WA	-1.09
HSI00000814	Peacehealth	Vancouver	WA	-1.23
HSI00001109	Trios Health	Kennewick	WA	-1.33
HSI00000372	Fort HealthCare	Fort Atkinson	WI	-1.03
HSI00000428	Gundersen Health System	La Crosse	WI	-1.37
HSI00000699	Monroe Clinic	Monroe	WI	-1.66
HSI00000206	Cheyenne Regional	Cheyenne	WY	-1.05
HSI00001271	Wyoming Medical Center	Casper	WY	-1.21

© 2021 Segal et al. *JAMA Health Forum.*

Category 2

Health System ID	Name	City	State	Standardized Overuse Index
HSI00001116	UAB Health System	Birmingham	AL	-0.57
HSI00001166	University of Arkansas for Medical Sciences	Little Rock	AR	-0.96
HSI00000335	Eisenhower Medical Center	Rancho Mirage	CA	-0.96
HSI00000619	Marshall Medical Center	Placerville	CA	-0.54
HSI00000959	Santa Clara Valley Health and Hospital System	San Jose	CA	-0.59
HSI00001059	Tahoe Forest Health System	Truckee	CA	-0.54
HSI00000132	Boulder Community Health	Boulder	CO	-0.77
HSI00001032	Stamford Health	Stamford	CT	-0.67
HSI00000218	Christiana Care Health System	Wilmington	DE	-0.74
HSI00000509	Indian River Memorial Hospital	Vero Beach	FL	-0.97
HSI00000962	Sarasota Memorial Health Care System	Sarasota	FL	-0.53
HSI00001060	Tallahassee Memorial Healthcare	Tallahassee	FL	-0.65
HSI00001121	UF Health	Gainesville	FL	-0.68
HSI00000064	Augusta University Medical Center	Augusta	GA	-0.54
HSI00000454	Hawaii Health Systems Corporation	Honolulu	HI	-0.85
HSI00000666	Mercy Medical Center	Cedar Rapids	IA	-0.52
HSI00000934	Saint Lukes Health System	Boise	ID	-0.95
HSI00001074	The Carle Foundation	Urbana	IL	-0.81
HSI00000811	Parkview Health System	Fort Wayne	IN	-0.52
HSI00000109	Berkshire Health Systems	Pittsfield	MA	-0.53
HSI00000443	Hallmark Health System	Melrose	MA	-0.69
HSI00001219	Valley Health Systems	Holyoke	MA	-0.92
HSI00000155	Calvert Health System	Prince Frederick	MD	-0.92
HSI00000315	Dimensions Healthcare System	Cheverly	MD	-0.60
HSI00000531	Johns Hopkins Health System	Baltimore	MD	-0.95
HSI00000582	LifeBridge Health	Baltimore	MD	-0.60
HSI00001319	Holland Hospital	Holland	MI	-0.90
HSI00001177	University of Michigan Health System	Ann Arbor	MI	-0.64
HSI00000631	Mayo Clinic	Rochester	MN	-0.81
HSI00000381	Freeman Health System	Joplin	MO	-0.59
HSI00000705	Mosaic Life Care	Saint Joseph	MO	-0.74
HSI00001110	Truman Medical Centers	Kansas City	MO	-0.84
HSI00000107	Benefis Healthcare System	Great Falls	MT	-0.51

© 2021 Segal et al. *JAMA Health Forum.*

HSI00000114	Billings Clinic	Billings	MT	-0.89
HSI00000042	Appalachian Regional Healthcare System	Boone	NC	-0.62
HSI00000323	Duke University Health System	Durham	NC	-0.87
HSI00000449	Harnett Health Systems	Dunn	NC	-0.77
HSI00000691	Mission Health System	Asheville	NC	-0.52
HSI00001008	Southeastern Health	Lumberton	NC	-0.62
HSI00001107	Trinity Health	Minot	ND	-0.50
HSI00000299	Dartmouth-Hitchcock	Lebanon	NH	-0.80
HSI00000558	LRG Healthcare	Laconia	NH	-0.97
HSI00001339	North Country Healthcare	Littleton	NH	-0.80
HSI00000502	Hunterdon Healthcare System	Flemington	NJ	-0.70
HSI00001288	Brookdale University Hospital And Medical Center	Brooklyn	NY	-0.65
HSI00000236	Columbia Memorial Health	Hudson	NY	-0.75
HSI00000362	Finger Lakes Health	Geneva	NY	-0.68
HSI00000711	Mount Sinai Health System	New York	NY	-0.82
HSI00000731	New York Presbyterian Healthcare System	New York	NY	-0.54
HSI00000798	Oswego Health	Oswego	NY	-0.83
HSI00001080	The Erie County Medical Center Corporation	Buffalo	NY	-0.76
HSI00000566	Lake Health	Painesville	OH	-0.98
HSI00001086	The Ohio State University Wexner Medical Center	Columbus	OH	-0.56
HSI00000576	Legacy Health	Portland	OR	-0.93
HSI00001111	Tuality Healthcare	Hillsboro	OR	-0.57
HSI00000068	Avera Health	Sioux Falls	SD	-0.81
HSI00000958	Sanford Health	Sioux Falls	SD	-0.97
HSI00001165	University of Texas Health System	Austin	TX	-0.51
HSI00001186	University of Virginia Health System	Charlottesville	VA	-0.88
HSI00000900	Rutland Regional Health Services	Rutland	VT	-0.96
HSI00001185	University of Vermont Health Network	Burlington	VT	-0.84
HSI00001281	Astria Health System	Sunnyside	WA	-0.61
HSI00001133	UW Medicine	Seattle	WA	-0.51
HSI00001232	Virginia Mason Health System	Seattle	WA	-0.81
HSI00000057	Aspirus	Wausau	WI	-0.63
HSI00000106	Beloit Health System	Beloit	WI	-0.91
HSI00000667	Mercy Health	Janesville	WI	-0.77

© 2021 Segal et al. *JAMA Health Forum.*

Category 3

Health System ID	Name	City	State	Standardized Overuse Index
HSI00001180	University of South Alabama Hospitals	Mobile	AL	0.31
HSI00000911	Saint Bernards Healthcare	Jonesboro	AR	0.19
HSI00001240	Washington Regional	Fayetteville	AR	0.17
HSI00001260	White River Health System	Batesville	AR	-0.03
HSI00000073	Banner Health	Phoenix	AZ	0.29
HSI00000761	Northern Arizona Healthcare	Flagstaff	AZ	0.15
HSI00001055	TMC Healthcare	Tucson	AZ	-0.17
HSI00001275	Yuma Regional Medical Center	Yuma	AZ	0.50
HSI00000011	Adventist Health	Roseville	CA	-0.12
HSI00001278	American Academic Health System	El Segundo	CA	0.46
HSI00000086	Barton Healthcare System	South Lake Tahoe	CA	0.44
HSI00000190	Cedars-Sinai Health System	West Hollywood	CA	0.17
HSI00000255	Community Memorial Health System	Ventura	CA	-0.46
HSI00000530	John Muir Health	Walnut Creek	CA	0.16
HSI00000540	Kaweah Delta Health Care District	Visalia	CA	0.08
HSI00000591	Loma Linda University Adventist Health Sciences Center	Loma Linda	CA	0.16
HSI00000616	Marin Healthcare District	Greenbrae	CA	-0.15
HSI00000809	Palomar Health	Escondido	CA	-0.20
HSI00000845	Prime Healthcare Services	Ontario	CA	0.08
HSI00000924	Saint Joseph Health System	Irvine	CA	0.32
HSI00000949	Salinas Valley Memorial Healthcare System	Salinas	CA	0.39
HSI00000977	Sharp Healthcare	San Diego	CA	0.19
HSI00001033	Stanford Health Care	Stanford	CA	-0.49
HSI00001050	Sutter Health	Sacramento	CA	0.13
HSI00001099	Torrance Memorial Medical Center	Torrance	CA	0.13
HSI00001168	UC Health	Oakland	CA	-0.17
HSI00001181	University of Southern California	Los Angeles	CA	0.18
HSI00001224	Verity Health System	El Segundo	CA	-0.03
HSI00001239	Washington Hospital Healthcare System	Fremont	CA	-0.24
HSI00000183	Catholic Health Initiatives	Englewood	CO	0.29
HSI00000991	SCL Health	Broomfield	CO	-0.02
HSI00001169	University of Colorado Health	Aurora	CO	-0.49

© 2021 Segal et al. *JAMA Health Forum.*

HSI00001214	Vail Health	Vail	CO	-0.09
HSI00000425	Griffin Health Services	Derby	CT	-0.29
HSI00000452	Hartford Healthcare Corporation	Hartford	CT	-0.13
HSI00000683	Middlesex Health System	Middletown	CT	0.29
HSI00001170	University of Connecticut Health Center	Farmington	CT	-0.14
HSI00001253	Western Connecticut Health Network	Danbury	CT	0.44
HSI00001272	Yale New Haven Health System	New Haven	CT	0.31
HSI00000498	Howard University Hospital	Washington	DC	0.13
HSI00000094	Bayhealth	Dover	DE	-0.01
HSI00000718	Nanticoke Health Services	Seaford	DE	0.23
HSI00000010	Adventist Health System	Altamonte Springs	FL	0.11
HSI00000076	Baptist Health Care	Pensacola	FL	-0.44
HSI00000195	Central Florida Health Alliance	Leesburg	FL	0.26
HSI00000368	Florida Health Sciences Center	Tampa	FL	0.43
HSI00000523	Jackson Health System	Miami	FL	-0.04
HSI00000716	NCH Healthcare System	Naples	FL	-0.21
HSI00000812	Parrish Healthcare	Titusville	FL	0.20
HSI00001042	Success Healthcare	Boca Raton	FL	-0.35
HSI00000235	Colquitt Regional Medical Center	Moultrie	GA	0.18
HSI00000241	Columbus Regional Healthcare System	Columbus	GA	0.40
HSI00000342	Emory Healthcare	Atlanta	GA	0.31
HSI00000369	Floyd Medical Center	Rome	GA	0.37
HSI00000431	Gwinnett Health System	Lawrenceville	GA	-0.35
HSI00001330	Memorial Hospital and Manor	Bainbridge	GA	-0.09
HSI00000724	Navicent Health	Macon	GA	-0.21
HSI00000823	Phoebe Putney Health Systems	Albany	GA	-0.10
HSI00000826	Piedmont Healthcare	Atlanta	GA	0.47
HSI00000927	Saint Josephs Candler Health System	Savannah	GA	0.10
HSI00000999	South Georgia Medical Center	Valdosta	GA	-0.07
HSI00001097	Tift Regional Health System	Tifton	GA	0.43
HSI00000455	Hawaii Pacific Health	Honolulu	HI	-0.18
HSI00000554	Kuakini Health System	Honolulu	HI	0.04
HSI00001087	The Queens Health Systems	Honolulu	HI	0.09
HSI00000390	Genesis Health System	Davenport	IA	-0.42
HSI00001146	Unitypoint Health	West Des Moines	IA	-0.27
HSI00000071	Bingham Memorial Hospital	Blackfoot	ID	-0.32
HSI00000553	Kootenai Health	Coeur D Alene	ID	-0.42

© 2021 Segal et al. *JAMA Health Forum.*

HSI00001277	Advocate Aurora Health	Downers Grove	IL	-0.04
HSI00000013	Advocate Health Care	Downers Grove	IL	0.49
HSI00000117	Blessing Health System	Quincy	IL	0.49
HSI00000191	Centegra Health System	Crystal Lake	IL	0.13
HSI00000289	Covenant Ministries of Benevolence	Chicago	IL	0.42
HSI00000334	Edward Elmhurst Healthcare	Naperville	IL	-0.38
HSI00000382	Freeport Health Network	Freeport	IL	0.03
HSI00000496	Hospital Sisters Health System	Springfield	IL	0.44
HSI00000756	NorthShore University Health System	Evanston	IL	-0.30
HSI00000776	OSF Healthcare System	Peoria	IL	0.21
HSI00000897	Rush System for Health	Chicago	IL	-0.02
HSI00000961	Sarah Bush Lincoln Health System	Mattoon	IL	-0.27
HSI00000987	Sinai Health System	Chicago	IL	-0.09
HSI00001009	Southern Illinois Healthcare	Carbondale	IL	0.15
HSI00001089	The University of Chicago Medicine	Chicago	IL	-0.01
HSI00000240	Columbus Regional Health	Columbus	IN	0.42
HSI00000248	Community Health Network	Indianapolis	IN	-0.34
HSI00000370	Floyd Memorial Hospital and Health Services	New Albany	IN	0.38
HSI00000375	Franciscan Health	Mishawaka	IN	0.50
HSI00000405	Good Samaritan Hospital	Vincennes	IN	-0.28
HSI00001309	Goshen Health	Goshen	IN	0.48
HSI00001314	Hancock Health	Greenfield	IN	0.41
HSI00000472	Hendricks Regional Health	Danville	IN	0.28
HSI00000474	Henry County Hospital	New Castle	IN	-0.11
HSI00000510	Indiana University Health	Indianapolis	IN	0.23
HSI00000612	Major Health Partners	Shelbyville	IN	0.17
HSI00001352	Schneck Medical Center	Seymour	IN	-0.41
HSI00001082	The Methodist Hospitals	Gary	IN	0.36
HSI00001137	Union Health	Terre Haute	IN	0.31
HSI00001269	Witham Health Services	Lebanon	IN	0.07
HSI00000456	Hays Medical Center, Inc	Hays	KS	-0.11
HSI00000788	Olathe Health System	Olathe	KS	0.37
HSI00001173	The University of Kansas Health System	Kansas City	KS	-0.19
HSI00000041	Appalachian Regional Healthcare	Lexington	KY	-0.18
HSI00000056	Kings Daughters Medical Center	Ashland	KY	-0.38
HSI00000260	Methodist Hospital	Henderson	KY	0.01

© 2021 Segal et al. *JAMA Health Forum.*

HSI00000771	Norton Healthcare	Louisville	KY	0.15
HSI00000802	Owensboro Health	Owensboro	KY	0.42
HSI00000917	Saint Elizabeth Healthcare	Edgewood	KY	-0.21
HSI00001053	T J Regional Health	Glasgow	KY	0.01
HSI00001174	University of Kentucky Healthcare	Lexington	KY	-0.41
HSI00000089	Baton Rouge General Health System	Baton Rouge	LA	-0.12
HSI00000376	Franciscan Missionaries of Our Lady Health System	Baton Rouge	LA	-0.07
HSI00000562	Lafayette General Health	Lafayette	LA	-0.04
HSI00000564	Lake Charles Memorial Health System	Lake Charles	LA	0.13
HSI00000555	LCMC Health System	New Orleans	LA	-0.10
HSI00000780	Ochsner Health System	New Orleans	LA	-0.23
HSI00001263	Willis-Knighton Health System	Shreveport	LA	0.04
HSI00000037	Anna Jacques Hospital	Newburyport	MA	-0.03
HSI00000097	Baystate Health	Springfield	MA	0.04
HSI00000110	Beth Israel Deaconess Medical Center	Boston	MA	-0.21
HSI00000340	Emerson Hospital Health System	Concord	MA	0.47
HSI00000478	Heywood Healthcare	Gardner	MA	-0.41
HSI00000563	Lahey Health System	Burlington	MA	0.40
HSI00000598	Lowell General Hospital	Lowell	MA	0.48
HSI00000708	Mount Auburn Hospital	Cambridge	MA	0.16
HSI00000813	Partners Healthcare System	Boston	MA	0.08
HSI00000984	Signature Healthcare	Brockton	MA	0.35
HSI00001002	South Shore Health System	South Weymouth	MA	0.33
HSI00001122	UMass Memorial Health Care	Worcester	MA	-0.31
HSI00001245	Wellforce Health System	Burlington	MA	-0.48
HSI00000038	Anne Arundel Health System	Annapolis	MD	0.26
HSI00000125	Bon Secours Health System	Marriottsville	MD	0.30
HSI00000422	Greater Baltimore Medical Center	Baltimore	MD	0.45
HSI00000640	MedStar Health	Columbia	MD	0.27
HSI00000818	Peninsula Regional Health System	Salisbury	MD	-0.05
HSI00001136	Union Hospital	Elkton	MD	0.33
HSI00001175	University of Maryland Medical System	Baltimore	MD	0.41
HSI00001255	Western Maryland Health System	Cumberland	MD	0.32
HSI00000139	Bronson Healthcare	Kalamazoo	MI	0.20

© 2021 Segal et al. *JAMA Health Forum.*

HSI00000475	Henry Ford Health System	Detroit	MI	0.09
HSI00000570	Lakeland Regional Health System	Saint Joseph	MI	0.24
HSI00000636	McLaren Health Care Corporation	Grand Blanc	MI	0.07
HSI00000652	Memorial Healthcare	Owosso	MI	-0.24
HSI00000682	MidMichigan Health	Midland	MI	-0.07
HSI00000715	Munson Healthcare	Traverse City	MI	0.21
HSI00001018	Sparrow Health System	Lansing	MI	0.15
HSI00001021	Spectrum Health	Grand Rapids	MI	-0.30
HSI00001106	Trinity Health	Livonia	MI	-0.10
HSI00000029	Allina Health System	Minneapolis	MN	-0.07
HSI00000193	CentraCare Health System	Saint Cloud	MN	-0.33
HSI00000357	Fairview Health Services	Minneapolis	MN	-0.30
HSI00000463	HealthEast Care System	Saint Paul	MN	-0.09
HSI00000464	HealthPartners	Minneapolis	MN	-0.07
HSI00000567	Lake Region Healthcare	Fergus Falls	MN	0.21
HSI00000881	Ridgeview Medical Center	Waconia	MN	0.04
HSI00000055	Ascension Health	Saint Louis	MO	0.26
HSI00000070	BJC Healthcare	Saint Louis	MO	-0.20
HSI00000290	Cox Health	Springfield	MO	0.31
HSI00000568	Lake Regional Health System	Osage Beach	MO	-0.32
HSI00000660	Mercy Health	Chesterfield	MO	0.01
HSI00000909	Saint Anthony Medical Center	Saint Louis	MO	-0.47
HSI00000920	Saint Francis Healthcare System	Cape Girardeau	MO	-0.23
HSI00000935	Saint Lukes Health System	Kansas City	MO	0.09
HSI00001007	Southeast Health	Cape Girardeau	MO	0.05
HSI00000902	SSM Health	Saint Louis	MO	0.15
HSI00001178	University of Missouri Health Care	Columbia	MO	-0.18
HSI00000034	Anderson Regional Health System	Meridian	MS	-0.08
HSI00000371	Forrest Health	Hattiesburg	MS	-0.15
HSI00001313	Greenwood Leflore Hospital	Greenwood	MS	-0.38
HSI00000750	North Mississippi Health Services	Tupelo	MS	-0.19
HSI00000996	South Central Reg Medical Center	Laurel	MS	0.42
HSI00000254	Community Medical Center	Missoula	MT	-0.29
HSI00000173	Atrium Health	Charlotte	NC	0.24
HSI00000171	Caromont Health System	Gastonia	NC	-0.25
HSI00000182	Catawba Valley Medical Center	Hickory	NC	-0.29
HSI00001123	UNC Health Care System	Chapel Hill	NC	-0.22

© 2021 Segal et al. *JAMA Health Forum.*

HSI00001230	Vidant Health	Greenville	NC	0.03
HSI00001236	Wakemed Health and Hospitals	Raleigh	NC	-0.09
HSI00000032	Altru Health System	Grand Forks	ND	0.49
HSI00000143	Bryan Health	Lincoln	NE	0.24
HSI00000358	Faith Regional Health Services	Norfolk	NE	-0.15
HSI00001085	The Nebraska Medical Center	Omaha	NE	0.09
HSI00000164	Capital Health System	Trenton	NJ	0.22
HSI00000194	Centrastate Healthcare System	Freehold	NJ	0.03
HSI00000942	Saint Peters Healthcare System	New Brunswick	NJ	-0.03
HSI00001154	University Hospital	Newark	NJ	0.48
HSI00001233	Virtua Health	Marlton	NJ	-0.24
HSI00000956	San Juan Regional Medical Center	Farmington	NM	0.34
HSI00000874	Renown Health	Reno	NV	0.40
HSI00000186	Catholic Health Services of Long Island	Rockville Centre	NY	0.13
HSI00000189	Cayuga Medical Center	Ithaca	NY	0.21
HSI00000401	Glens Falls Hospital	Glens Falls	NY	-0.35
HSI00000423	Greater Hudson Valley Health System	Middletown	NY	0.19
HSI00000607	Maimonides Medical Center	Brooklyn	NY	0.00
HSI00000697	Mohawk Valley Health System	Utica	NY	-0.07
HSI00000700	Montefiore Medical Center	Bronx	NY	0.19
HSI00001335	Nathan Littauer Hospital Association	Gloversville	NY	0.07
HSI00000737	Niagara Falls Memorial Medical Center	Niagara Falls	NY	0.30
HSI00000767	Northwell Health	New Hyde Park	NY	-0.15
HSI00000717	NYU Langone Health	New York	NY	0.04
HSI00000879	Richmond University Medical Center	Staten Island	NY	0.15
HSI00000889	Rochester Regional Health System	Rochester	NY	0.27
HSI00000925	Saint Joseph Medical Center	Yonkers	NY	0.33
HSI00000930	Saint Lawrence Health System	Potsdam	NY	0.43
HSI00000932	Saint Lukes Cornwall Hospital	Newburgh	NY	-0.09
HSI00000952	Samaritan Health System	Watertown	NY	-0.26
HSI00000963	Saratoga Hospital	Saratoga Springs	NY	0.23
HSI00000904	SUNY Upstate Medical University	Syracuse	NY	0.04
HSI00001138	United Health Services	Binghamton	NY	0.13
HSI00001179	University of Rochester Medical Center	Rochester	NY	-0.44

© 2021 Segal et al. *JAMA Health Forum.*

HSI00000065	Aultman Health Foundation	Canton	OH	-0.29
HSI00000356	Fairfield Medical Center	Lancaster	OH	-0.28
HSI00000363	Firelands Regional Health System	Sandusky	OH	-0.15
HSI00000391	Genesis Healthcare System	Zanesville	OH	0.18
HSI00000581	Licking Memorial Health Systems	Newark	OH	-0.08
HSI00000649	Memorial Health System	Marietta	OH	0.29
HSI00000661	Mercy Health	Cincinnati	OH	0.25
HSI00000784	Ohiohealth	Columbus	OH	0.36
HSI00000990	Sisters of Charity Health System	Cleveland	OH	-0.29
HSI00001078	The Cleveland Clinic Health System	Cleveland	OH	-0.09
HSI00001104	TriHealth	Cincinnati	OH	0.40
HSI00001108	Trinity Health System	Steubenville	OH	0.39
HSI00001156	University Hospitals	Shaker Heights	OH	-0.44
HSI00000242	Comanche County Memorial Hospital	Lawton	OK	-0.06
HSI00000743	Norman Regional Health System	Norman	OK	0.46
HSI00001359	Stillwater Medical Center Authority	Stillwater	OK	-0.39
HSI00000054	Asante Health System	Medford	OR	-0.26
HSI00000681	Mid Columbia Medical Center	The Dalles	OR	-0.26
HSI00000795	Oregon Health and Science University	Portland	OR	-0.15
HSI00000994	Sky Lakes Medical Center	Klamath Falls	OR	-0.17
HSI00000020	Albert Einstein Healthcare Network	Philadelphia	PA	0.25
HSI00000388	Geisinger Health System	Danville	PA	0.12
HSI00000430	Guthrie Healthcare System	Sayre	PA	0.49
HSI00000476	Heritage Valley Health System	Beaver	PA	0.48
HSI00000488	Holy Redeemer Health System	Huntingdon Valley	PA	0.17
HSI00000686	Lecom Health	Erie	PA	-0.06
HSI00000577	Lehigh Valley Health Network	Allentown	PA	0.39
HSI00000639	Meadville Medical Center	Meadville	PA	0.25
HSI00000819	Penn Highlands Healthcare	Du Bois	PA	0.15
HSI00000821	Penn State Milton S Hershey Medical Center	Hershey	PA	-0.09
HSI00000936	Saint Lukes University Health Network	Bethlehem	PA	0.32
HSI00001045	Summit Health	Chambersburg	PA	0.45
HSI00001049	Susquehanna Health System	Williamsport	PA	-0.26
HSI00000820	University of Pennsylvania Health System	Philadelphia	PA	0.25

© 2021 Segal et al. *JAMA Health Forum.*

HSI00000202	Chartercare Health Partners	Providence	RI	-0.21
HSI00000585	Lifespan	Providence	RI	0.17
HSI00000100	Beaufort Memorial Hospital	Beaufort	SC	0.21
HSI00000424	Greenville Health System	Greenville	SC	0.25
HSI00001020	Spartanburg Regional Healthcare System	Spartanburg	SC	-0.21
HSI00000046	Ardent Health Services	Nashville	TN	0.47
HSI00001284	Ballad Health	Johnson City	TN	0.24
HSI00000081	Baptist Memorial Health Care Corporation	Memphis	TN	0.27
HSI00000118	Blount Memorial Hospital	Maryville	TN	-0.44
HSI00000295	Curae Health	Clinton	TN	0.45
HSI00000203	Erlanger Health System	Chattanooga	TN	-0.12
HSI00000630	Maury Regional Medical Center	Columbia	TN	0.48
HSI00000675	Methodist Le Bonheur Healthcare	Memphis	TN	0.31
HSI00000713	Mountain States Health Alliance	Johnson City	TN	-0.12
HSI00000096	Baylor Scott and White Health	Dallas	TX	0.25
HSI00000152	Christus Health	Irving	TX	0.13
HSI00000251	Community Hospital Corporation	Plano	TX	0.30
HSI00000328	East Texas Medical Center Regional Healthcare System	Tyler	TX	0.12
HSI00000471	Hendrick Health System	Abilene	TX	0.12
HSI00000557	LHP Hospital Group, Inc	Plano	TX	0.24
HSI00000747	North Cypress Medical Center	Cypress	TX	0.32
HSI00001143	United Regional Health Care System	Wichita Falls	TX	0.33
HSI00000113	University Health System	San Antonio	TX	-0.14
HSI00001159	University Medical Center Health System	Lubbock	TX	-0.10
HSI00000516	Intermountain Healthcare	Salt Lake City	UT	0.12
HSI00000170	Carilion Clinic	Roanoke	VA	-0.12
HSI00000192	Centra Health	Lynchburg	VA	-0.14
HSI00000513	Inova Health System	Falls Church	VA	0.39
HSI00000624	Mary Washington Healthcare	Fredericksburg	VA	0.37
HSI00000883	Riverside Health System	Newport News	VA	-0.22
HSI00000972	Sentara Healthcare	Norfolk	VA	-0.16
HSI00001217	Valley Health System	Winchester	VA	-0.29
HSI00001213	VCU Health System	Richmond	VA	-0.38
HSI00001016	Southwestern Vermont Health Care	Bennington	VT	-0.47
HSI00001022	Springfield Medical Care	Springfield	VT	0.06

© 2021 Segal et al. *JAMA Health Forum.*

	Systems			
HSI00000427	Group Health Cooperative	Seattle	WA	0.23
HSI00001356	Island Hospital	Anacortes	WA	0.37
HSI00000852	Providence Saint Joseph Health	Renton	WA	0.10
HSI00001051	Swedish Health Services	Seattle	WA	-0.02
HSI00000015	Agnesian Healthcare	Fond Du Lac	WI	-0.06
HSI00000105	Bellin Health Systems	Green Bay	WI	0.30
HSI00000386	Froedtert and The Medical College of Wisconsin	Milwaukee	WI	-0.29
HSI00000487	Holy Family Memorial	Manitowoc	WI	-0.40
HSI00000621	Marshfield Clinic	Marshfield	WI	0.20
HSI00000847	Prohealth Care	Waukesha	WI	0.05
HSI00001132	UW Health	Madison	WI	-0.42
HSI00000698	Mon Health	Morgantown	WV	0.31
HSI00000940	Saint Marys Medical Management	Huntington	WV	-0.36
HSI00001093	Thomas Health System	Charleston	WV	0.28
HSI00001251	West Virginia United Health System	Morgantown	WV	0.31
HSI00000159	Campbell County Health	Gillette	WY	-0.10

© 2021 Segal et al. *JAMA Health Forum.*

Category 4

Health System ID	Name	City	State	Standardized Overuse Index
HSI00000297	DCH Health System	Tuscaloosa	AL	0.54
HSI00000467	Huntsville Hospital Health System	Huntsville	AL	0.65
HSI00000074	Baptist Health	Little Rock	AR	0.52
HSI00000090	Baxter Regional Medical Center	Mountain Home	AR	0.81
HSI00000491	HonorHealth	Scottsdale	AZ	0.61
HSI00001273	Yavapai Regional Medical Center	Prescott	AZ	0.83
HSI00000021	Alecto Healthcare Services	Irvine	CA	0.76
HSI00000067	Avanti Hospitals	El Segundo	CA	0.77
HSI00000226	Citrus Valley Health Partners	Covina	CA	0.92
HSI00000283	Cottage Health System	Santa Barbara	CA	0.89
HSI00000314	Dignity Health	San Francisco	CA	0.55
HSI00000484	Hoag Memorial Hospital Presbyterian	Newport Beach	CA	0.51
HSI00000755	Northbay Healthcare Group	Fairfield	CA	0.73
HSI00000833	Pomona Valley Hospital Medical Center	Pomona	CA	0.60
HSI00000851	Prospect Medical Holdings	Los Angeles	CA	0.52
HSI00000954	San Antonio Regional Hospital	Upland	CA	0.79
HSI00000968	Scripps Health	San Diego	CA	0.72
HSI00001341	Parkview Medical Center	Pueblo	CO	0.62
HSI00001365	Valley View Hospital Association	Glenwood Springs	CO	0.89
HSI00001286	Bristol Hospital	Bristol	CT	1.00
HSI00000301	Day Kimball Healthcare	Putnam	CT	0.83
HSI00000574	Lawrence and Memorial Hospital	New London	CT	0.94
HSI00000589	Little Company of Mary Hospital and Health Care Centers	Evergreen Park	CT	0.83
HSI00000075	Baptist Health	Jacksonville	FL	0.57
HSI00000112	Bethesda Health, Inc	Boynton Beach	FL	0.87
HSI00000461	Health First	Rockledge	FL	0.78
HSI00000575	Lee Health	Fort Myers	FL	0.71
HSI00000653	Memorial Healthcare System	Hollywood	FL	0.94
HSI00000712	Mount Sinai Medical Center of Florida	Miami Beach	FL	0.86
HSI00000796	Orlando Health	Orlando	FL	0.77
HSI00000045	Archbold Medical Center	Thomasville	GA	0.89
HSI00000497	Houston Healthcare	Warner Robins	GA	0.76
HSI00000651	Memorial Health University Medical Center	Savannah	GA	0.98
HSI00001006	Southeast Georgia Health System	Brunswick	GA	0.55
HSI00001061	Tanner Health System	Carrollton	GA	0.68

© 2021 Segal et al. *JAMA Health Forum.*

HSI00001244	WellStar Health System	Marietta	GA	0.74
HSI00000665	Mercy Iowa City	Iowa City	IA	0.54
HSI00000648	Memorial Health System	Springfield	IL	0.70
HSI00000768	Northwest Community Healthcare	Arlington Heights	IL	0.88
HSI00000770	Northwestern Medicine	Chicago	IL	0.88
HSI00001340	Palos Health	Palos Heights	IL	0.63
HSI00000844	Presence Health	Chicago	IL	0.86
HSI00001084	The Morris Hospital and Healthcare Centers	Morris	IL	0.91
HSI00000098	Beacon Health System	South Bend	IN	0.58
HSI00001073	Kings Daughters Health	Madison	IN	0.78
HSI00000873	Reid Health	Richmond	IN	0.70
HSI00000887	Riverview Health	Noblesville	IN	0.60
HSI00001039	Stormont Vail Health	Topeka	KS	0.94
HSI00000080	Baptist Healthcare System	Louisville	KY	0.88
HSI00000244	Commonwealth Health Corporation	Bowling Green	KY	0.76
HSI00000326	East Jefferson General Hospital	Metairie	LA	0.93
HSI00000751	North Oaks Health System	Hammond	LA	0.92
HSI00000528	West Jefferson Medical Center	Marrero	LA	0.65
HSI00000161	Cape Cod Healthcare	Hyannis	MA	0.79
HSI00001041	Sturdy Memorial Hospital	Attleboro	MA	0.50
HSI00000012	Adventist Healthcare	Gaithersburg	MD	0.56
HSI00000059	Atlantic General Health System	Berlin	MD	0.56
HSI00000101	Beaumont Health Systems	Southfield	MI	0.95
HSI00001188	Upper Peninsula Health Care Solutions	Marquette	MI	0.52
HSI00000258	Community Memorial Hospital Association	Cloquet	MN	0.61
HSI00000749	North Memorial Health Care	Robbinsdale	MN	0.79
HSI00000933	Saint Lukes Episcopal Presbyterian Hospital	Chesterfield	MO	0.76
HSI00000692	Mississippi Baptist Health Systems, Inc	Jackson	MS	0.81
HSI00000896	Rush Health Systems	Meridian	MS	0.53
HSI00000988	Singing River Health System	Gautier	MS	0.82
HSI00001155	University of Mississippi Health Care	Jackson	MS	0.55
HSI00000538	Kalispell Regional Healthcare	Kalispell	MT	0.73
HSI00000231	Coastal Carolinas Health Alliance	Wilmington	NC	0.69
HSI00000263	Cone Health	Greensboro	NC	0.79
HSI00000365	Firsthealth of The Carolinas	Pinehurst	NC	0.63
HSI00000773	Novant Health	Winston Salem	NC	0.55
HSI00000753	Great Plains Health	North Platte	NE	0.55
HSI00000672	Methodist Health System	Omaha	NE	0.74
HSI00000871	Regional West Health Services	Scottsbluff	NE	0.58

© 2021 Segal et al. *JAMA Health Forum.*

HSI00000355	Exeter Health Resources	Exeter	NH	0.78
HSI00001358	Southern New Hampshire Health	Nashua	NH	0.62
HSI00000060	Atlantic Health System	Morristown	NJ	0.65
HSI00000439	Hackensack Meridian Health	Edison	NJ	0.76
HSI00000514	Inspira Health Network	Mullica Hill	NJ	0.79
HSI00000521	JFK Health System	Edison	NJ	0.60
HSI00000541	Kennedy Health System	Voorhees	NJ	0.55
HSI00000863	RWJBarnabas Health	West Orange	NJ	0.66
HSI00000929	Saint Josephs Healthcare System	Paterson	NJ	0.79
HSI00001079	The Cooper Health System	Camden	NJ	0.61
HSI00001105	Trinitas Regional Medical Center	Elizabeth	NJ	0.62
HSI00001218	Valley Health System	Ridgewood	NJ	0.95
HSI00000178	Carson Tahoe Health	Carson City	NV	0.61
HSI00000019	Albany Medical Center	Albany	NY	0.90
HSI00000052	Arnot Health	Elmira	NY	0.56
HSI00000293	Crouse Health System	Syracuse	NY	0.83
HSI00000462	Health Quest	Lagrangeville	NY	0.64
HSI00000592	Long Island Health Network	Melville	NY	0.55
HSI00001001	South Nassau Communities Hospital	Oceanside	NY	0.65
HSI00001038	Stony Brook Medicine	Stony Brook	NY	0.61
HSI00001252	Westchester Medical Center	Valhalla	NY	0.50
HSI00000108	Berger Health System	Circleville	OH	0.73
HSI00000367	Fisher Titus Medical Center	Norwalk	OH	0.70
HSI00000848	ProMedica Health System	Toledo	OH	0.66
HSI00001077	The Christ Hospital Health Network	Cincinnati	OH	0.75
HSI00000507	Integris Health	Oklahoma City	OK	0.61
HSI00000919	Saint Francis Health System	Tulsa	OK	0.83
HSI00000051	Armstrong County Memorial Hospital	Kittanning	PA	0.69
HSI00000354	Excela Health	Greensburg	PA	0.63
HSI00000048	Jefferson Health	Radnor	PA	0.93
HSI00000608	Main Line Health	Bryn Mawr	PA	0.51
HSI00000828	PinnacleHealth System	Harrisburg	PA	0.75
HSI00000913	Saint Clair Memorial Hospital	Pittsburgh	PA	0.97
HSI00000866	Tower Health	West Reading	PA	0.91
HSI00001148	Universal Health Services	Norristown	PA	0.82
HSI00001126	UPMC	Pittsburgh	PA	0.51
HSI00001238	Washington Health System	Washington	PA	0.86
HSI00001243	Wellspan Health	York	PA	0.52
HSI00000998	South County Health	Wakefield	RI	0.94
HSI00000271	Conway Medical Center	Conway	SC	0.74
HSI00000637	McLeod Health	Florence	SC	0.92

© 2021 Segal et al. *JAMA Health Forum.*

HSI00001354	Self Regional Healthcare	Greenwood	SC	0.81
HSI00001096	Tidelands Health	Georgetown	SC	0.81
HSI00000249	Community Health Systems	Franklin	TN	0.61
HSI00000275	Cookeville Regional Health System	Cookeville	TN	0.54
HSI00000286	Covenant Health	Knoxville	TN	0.70
HSI00000492	HCA Healthcare	Nashville	TN	0.57
HSI00000506	IASIS Healthcare Corporation	Franklin	TN	0.78
HSI00000584	Lifepoint Health	Brentwood	TN	0.50
HSI00000862	RCCH HealthCare Partners	Brentwood	TN	0.56
HSI00001153	University of Tennessee Medical Center	Knoxville	TN	0.51
HSI00001246	Wellmont Health System	Kingsport	TN	0.63
HSI00001250	West Tennessee Healthcare	Jackson	TN	0.56
HSI00000406	Good Shepherd Health System	Longview	TX	0.79
HSI00000643	Medical Center Health System	Odessa	TX	0.59
HSI00001037	Steward Health Care System	Dallas	TX	0.74
HSI00001066	Tenet Healthcare Corporation	Dallas	TX	0.79
HSI00001072	Texas Health Resources	Arlington	TX	0.97
HSI00000066	Aurora Health Care, Inc	Milwaukee	WI	0.70
HSI00001092	ThedaCare	Appleton	WI	0.92
HSI00000154	Cabell Huntington Hospital	Huntington	WV	0.71
HSI00001259	Wheeling Hospital	Wheeling	WV	0.85

© 2021 Segal et al. *JAMA Health Forum.*

Category 5

Health System ID	Name	City	State	Standardized Overuse Index
HSI00000466	Baptist Health	Montgomery	AL	1.30
HSI00000325	East Alabama Medical Center	Opelika	AL	1.97
HSI00000511	Infirmary Health System	Mobile	AL	1.11
HSI00001005	Southeast Health	Dothan	AL	1.80
HSI00000272	Conway Regional Health System	Conway	AR	1.20
HSI00000253	Community Medical Centers	Clovis	CA	2.33
HSI00000336	El Camino Hospital	Mountain View	CA	1.50
HSI00000404	Good Samaritan Hospital	Los Angeles	CA	1.23
HSI00000658	MemorialCare Health System	Fountain Valley	CA	1.06
HSI00000805	PIH Health	Whittier	CA	1.21
HSI00000329	Eastern Connecticut Health Network	Manchester	CT	1.19
HSI00000103	Beebe Healthcare	Lewes	DE	1.36
HSI00000077	Baptist Health South Florida	Coral Gables	FL	1.08
HSI00000121	Boca Raton Regional Hospital	Boca Raton	FL	1.49
HSI00000745	Broward Health	Fort Lauderdale	FL	1.32
HSI00000441	Halifax Health	Daytona Beach	FL	1.09
HSI00000569	Lakeland Regional Health System	Lakeland	FL	1.60
HSI00000622	Martin Health System	Stuart	FL	1.01
HSI00000304	DeKalb Medical Center, Inc	Decatur	GA	1.25
HSI00000444	Hamilton Health Care System	Dalton	GA	1.33
HSI00000759	Northeast Georgia Health System	Gainesville	GA	1.53
HSI00000766	Northside Hospital	Atlanta	GA	1.05
HSI00001151	University Health Care System	Augusta	GA	1.94
HSI00000421	Great River Health Systems	West Burlington	IA	1.31
HSI00000985	Silver Cross Hospital	New Lenox	IL	1.20
HSI00000246	Community Foundation of Northwest Indiana	Munster	IN	1.13

© 2021 Segal et al. *JAMA Health Forum.*

HSI00000305	Deaconess Health System	Evansville	IN	1.92
HSI00000948	Salina Regional Health Center	Salina	KS	1.38
HSI00000346	Ephraim Mcdowell Health	Danville	KY	1.11
HSI00000944	Slidell Memorial Hospital	Slidell	LA	1.61
HSI00001004	Southcoast Health System	New Bedford	MA	1.36
HSI00000663	Mercy	Baltimore	MD	1.13
HSI00000671	Meritus Health	Hagerstown	MD	1.09
HSI00000288	Covenant HealthCare	Saginaw	MI	1.17
HSI00000505	Hurley Medical Center	Flint	MI	1.11
HSI00000729	New Liberty Hospital District of Clay County	Pleasant Valley	MO	1.15
HSI00000748	North Kansas City Hospital	North Kansas City	MO	1.59
HSI00000916	Saint Dominic Health Services	Jackson	MS	3.11
HSI00000172	CarolinaEast Health System	New Bern	NC	1.72
HSI00000518	Iredell Health System	Statesville	NC	1.88
HSI00000728	New Hanover Regional Medical Center	Wilmington	NC	1.21
HSI00000623	Mary Lanning Healthcare	Hastings	NE	1.52
HSI00000169	Carepoint Health	Jersey City	NJ	1.27
HSI00000267	Congregation Of The Sisters Of St Joseph Of Peace	Englewood Cliffs	NJ	1.91
HSI00000846	Princeton HealthCare System	Plainsboro	NJ	1.09
HSI00000339	Ellis Medicine	Schenectady	NY	1.19
HSI00000347	Episcopal Health Services	Bethpage	NY	1.39
HSI00000537	Kaleida Health	Buffalo	NY	1.51
HSI00000792	Oneida Healthcare	Oneida	NY	1.07
HSI00000882	Riverside Health Care System	Yonkers	NY	1.03
HSI00001187	Upper Allegheny Health System	Olean	NY	1.51
HSI00000546	Kettering Health Network	Kettering	OH	1.04
HSI00000842	Premier Health	Dayton	OH	1.05

© 2021 Segal et al. *JAMA Health Forum.*

HSI00000025	Allegheny Health Network	Pittsburgh	PA	1.49
HSI00000146	Butler Health System	Butler	PA	1.73
HSI00000320	Doylestown Hospital	Doylestown	PA	1.24
HSI00000352	Evangelical Community Hospital	Lewisburg	PA	1.13
HSI00000415	Grand View Hospital	Sellersville	PA	1.06
HSI00001324	Indiana Regional Medical Center	Indiana	PA	1.54
HSI00000710	Mount Nittany Health System	State College	PA	1.22
HSI00000832	Pocono Health System	East Stroudsburg	PA	1.97
HSI00000906	Sacred Heart HealthCare System	Allentown	PA	1.83
HSI00001065	Temple University Health System	Philadelphia	PA	1.56
HSI00000166	Care New England Health System	Providence	RI	2.14
HSI00000579	Lexington Medical Center	West Columbia	SC	1.35
HSI00000644	MUSC Health	Charleston	SC	1.28
HSI00000807	Palmetto Health	Columbia	SC	1.03
HSI00000318	Doctors Hospital at Renaissance	Edinburg	TX	1.28
HSI00001081	Houston Methodist	Houston	TX	1.00
HSI00000654	Memorial Hermann Healthcare System	Houston	TX	1.05
HSI00000673	Methodist Health System	Dallas	TX	1.11
HSI00000684	Midland Health	Midland	TX	1.03
HSI00000975	Shannon Health System	San Angelo	TX	1.41
HSI00001130	USMD Health System	Irving	TX	2.66
HSI00000205	Chesapeake Regional Medical Center	Chesapeake	VA	1.77
HSI00000147	CAMC Health System	Charleston	WV	1.10
HSI00000783	Ohio Valley Health Services and Education Corporation	Wheeling	WV	1.63

© 2021 Segal et al. *JAMA Health Forum.*

eFigure. Sensitivity Analysis with Exclusion of Low Count Hospitals

After exclusion of hospitals from contributing to an indicator if there were not 20 eligible individuals, the Standardized Overuse Index differed little. The plot below excludes one outlier with a very low Standardized Overuse Index

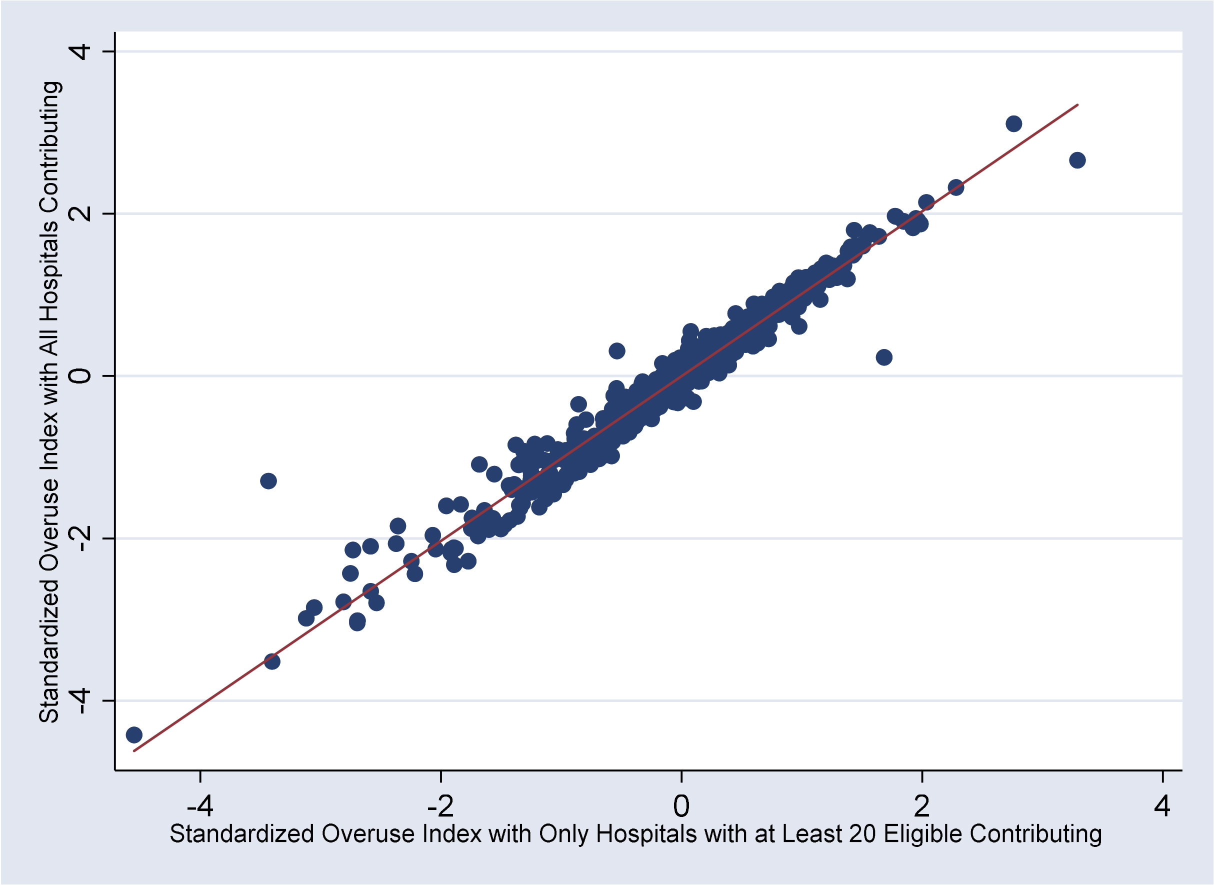

© 2021 Segal et al. *JAMA Health Forum.*

eTable 5. Tabulation of Health Systems with Inclusion of All Hospitals or Low Counts of Eligible Exclusions

Focusing on the most overusing categories (4 and 5), only 1 health system changed 2 categories, moving from category 3 to category 5, with the exclusion of hospitals with few eligible. Three health systems moved from category 4 to category 5 with exclusion of low count hospitals and 13 health systems moved from category 5 to 4 with exclusion of small hospitals.

Categories of Overuse with Exclusion of Hospitals with <20 Eligible

Categories of Overuse with All Hospitals Contributing

	1	2	3	4	5	Total
1	83	18	0	0	0	101
2	6	43	18	0	0	67
3	0	7	277	9	1	294
4	0	0	20	114	3	137
5	0	0	0	13	64	77

© 2021 Segal et al. *JAMA Health Forum.*

eTable 6. Independent Association of Health System Characteristics with (Standardized) Overuse Index with Inclusion of Random Effects for State

Characteristic	Category	N	Model 1. Change in Overuse Index* (N=486)		Model 2. Change in Overuse Index* (N=675)	
				P-value		P-value
**Primary care physician category**	Reference	227				
	2nd tertile	223	**-0.28**	**0.021**	**-0.33**	**0.004**
	3rd tertile	225	**-0.66**	**0.000**	**-0.70**	**0.000**
**Hospital count category**	Reference	259				
	2nd tertile	233	**0.19**	**0.043**	**0.23**	**0.009**
	3rd tertile	184	0.012	0.92	0.079	0.56
**Medical group count category**	Reference	235				
	2nd tertile	217	**0.27**	**0.015**	0.18	0.072
	3rd tertile	223	**0.38**	**0.012**	0.30	0.044
**Bed count category**	Reference	226				
	2nd tertile	225	0.13	0.23	**0.24**	**0.018**
	3rd tertile	225	**0.52**	**0.001**	**0.69**	**0.000**
**Teaching intensity**	Ref	212				
	Minor teaching	316	-0.10	0.32	-0.075	0.432
	Major teaching	148	**-0.43**	**0.002**	**-0.45**	**0.001**
**Is primarily investor owned**		20	**0.56**	**0.007**	0.25	0.22
**Includes a very major teaching hospital**		**104**	**-0.27**	**0.025**	**-0.28**	**0.032**
**Upper quartile of uncompensated care**		136	-0.39	0.097	**.**	**.**
**Participates in a Medicare bundled payment**		287	0.15	0.079	.	.
**Participates in a Medicare alternative payment model**		431	0.15	0.10	.	.

© 2021 Segal et al. *JAMA Health Forum.*

**Owns a Medicare Advantage plan**	110	-0.022	0.11	.	.
**Owns a Medicaid managed care plan**	96	-0.18	0.11	.	.
**Participates in an Accountable Care Organization contract**	283	0.049	0.093	.	.

*model includes random effects for primary state of the health system, all displayed variables are included in the model, the overuse index is standardized so a change of 1 reflects 1 standard deviation change, reference group is "no" for binary categories; absence of results means that this information was not available for all health systems; bold indicates statistical significant change with p-value≤0.05

© 2021 Segal et al. *JAMA Health Forum.*
